# Addition of anti‐PD‐1 immunotherapy to BRAF inhibitor‐based targeted therapy improves real‐world survival and delays brain metastases in patients with BRAF^V600^‐mutant advanced melanoma: a multicenter cohort study

**DOI:** 10.1002/mco2.70102

**Published:** 2025-02-17

**Authors:** Junwan Wu, Qiuyue Ding, Qiong Zhang, Qianqi Chen, Xizhi Wen, Ya Ding, Jingjing Li, Ziluan Chen, Tao Zhang, Jiuhong Wang, Fuxue Huang, Hang Jiang, Linbin Chen, Qiming Zhou, Ke Li, Xiaoshi Zhang, Dandan Li

**Affiliations:** ^1^ Biotherapy Center, Sun Yat‐sen University Cancer Center, State Key Laboratory of Oncology in South China, Guangdong Provincial Clinical Research Center for Cancer, Collaborative Innovation Center for Cancer Medicine Guangzhou Guangdong Province P. R. China; ^2^ State Key Laboratory of Oncology in South China, Guangdong Provincial Clinical Research Center for Cancer, Collaborative Innovation Center for Cancer Medicine, Sun Yat‐sen University Cancer Center Guangzhou Guangdong Province P. R. China; ^3^ Department of Oncology Huazhong University of Science and Technology Union Shenzhen Hospital Shenzhen Guangdong Province P. R. China; ^4^ Department of Cancer Biotherapy Center Yunnan Cancer Hospital, The Third Affiliated Hospital of Kunming Medical University, Cancer Center of Yunnan Province Kunming Yunnan Province P. R. China

**Keywords:** anti‐PD‐1, BRAF, brain metastasis, melanoma, targeted therapy

## Abstract

Anti‐PD‐1 immunotherapy and targeted therapy (TT) represent two major therapeutic modalities for BRAF^V600^‐mutant advanced melanoma, but the efficacy of combination therapy in Asian populations remains unknown. Asian melanoma patients differ significantly from Caucasians in tissue subtypes, pathogenesis and response to treatment. We retrospectively analyzed data of BRAF^V600^‐mutant advanced melanoma patients treated with first‐line vemurafenib (V) ± anti‐PD‐1 or dabrafenib+trametinib (D+T) ± anti‐PD‐1 between 2014 and 2023 from three centers in China. 178 patients were included, with V (*n* = 45), D+T (*n* = 51), V+anti‐PD‐1 (*n* = 39) and D+T+anti‐PD‐1 (*n* = 43). The median PFS (21.9 vs. 11.1 months, *p* < 0.001), OS (NR vs. 32.6 months, *p* = 0.027), and DoR (20.0 vs. 8.4 months, *p* = 0.002) were significantly prolonged with D+T+anti‐PD‐1 versus D+T. Addition of anti‐PD‐1 to V also significantly prolonged PFS, OS, and DoR (*p* < 0.001). V+anti‐PD‐1 was superior to D+T in terms of PFS (15.0 vs. 11.1 months, *p* = 0.007) and DoR (18.0 vs. 8.4 months, *p* = 0.013), and was comparable to D+T+anti‐PD‐1. Addition of anti‐PD‐1 to BRAF inhibitor‐based TT was associated with lower incidence of brain metastases (*p* = 0.032). Addition of anti‐PD‐1 to BRAF inhibitor‐based TT appears to be a safe and effective treatment option, conferring a survival benefit and delaying the onset brain metastases in patients with BRAF^V600^‐mutant advanced melanoma.

## Graphical abstract

‐ Vemurafenib combined with anti‐PD‐1 antibody seemed to be an effective alternative treatment.

‐ Brain was the most common new metastatic site at first recorded failure.

‐ Addition of anti‐PD‐1 antibody to BRAF inhibitor‐based targeted therapy delayed brain metastases.

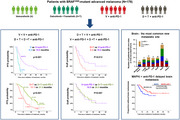

## 1 INTRODUCTION

Melanoma arises from melanocytes and is the most aggressive and deadly skin cancer.^1^, ^2^ Mutations in BRAF, the most important driver of cutaneous melanoma, are present in 40–50% of melanoma patients.^3^ In China, approximately 25% of melanoma patients carry BRAF mutations, of which more than 90% occur at V600, mostly V600E.^4^ Although BRAF inhibitor (BRAFi), including vemurafenib (V), dabrafenib (D), and encorafenib led to a high response rate, patients treated with BRAFi monotherapy were prone to develop resistance.^5^, ^6^, ^7^, ^8^ Improving upon the efficacy of BRAFi, the combination of BRAFi and MEK inhibitor (MEKi), such as D+trametinib (D+T) and V+cobimetinib, have significantly delayed resistance and improved survival.^9^, ^10^, ^11^, ^12^ In addition to targeted therapies (TT), the use of immune checkpoint blockade (ICB) against programmed cell death‐1 (PD‐1) protein or cytotoxic T lymphocyte antigen‐4 (CTLA‐4) has also greatly improved the treatment of advanced melanoma, with notable efficacy and long‐term survival benefits.^13^, ^14^, ^15^, ^16^

Although TT often demonstrates effectiveness in most patients, acquired resistance is a common occurrence.^17^ In contrast, patients have a lower response rate to ICB, but the response is more durable.^18^ Given these complementary features, the combination of the two approaches may prove beneficial. Moreover, their combination has been shown to increase immune activity in mouse models and enable CD4^+^ and CD8^+^ T cells to enter tumors in preclinical studies.^19^, ^20^, ^21^ Currently, a series of trials have been conducted to explore the superiority of the combination treatment. IMspire150 study was the only clinical trial to show a statistically significant progression‐free survival (PFS), whereas KEYNOTE‐022 and COMBI‐i studies did not reach the pre‐specified primary endpoint. Currently, the evidence supporting the advantages of combination therapy over TT alone in the Caucasian population is not overwhelmingly positive and somewhat controversial.^22^, ^23^, ^24^, ^25^, ^26^, ^27^ This field of study appears to be in its relative infancy, thus emphasizing the pressing necessity for further exploration and validation.

As we know, clinical trials investigating the efficacy and safety of combination therapy with anti‐PD‐1 and TT have all been conducted in Caucasian populations, and there is currently a lack of clinical trials and real‐world data in Asian populations. There are notable differences between Asian and Caucasian melanoma patients with regard to tissue subtypes, pathogenesis, and response to treatment.^28^, ^29^ It has been reported to be less sensitive to ICB than Caucasians and may have stronger primary resistance to ICB mainly due to a poorer immune microenvironment, lower PD‐L1 expression and lower tumor mutation load.^28^, ^29^ Therefore, for Chinese patients with BRAF^V600^‐mutant advanced melanoma, BRAFi‐based TT has been the preferred treatment recommended by Chinese Society of Clinical Oncology (CSCO) guideline, and we hypothesized that addition of anti‐PD‐1 to TT might prove to be a better treatment option.

The objective of the present study was to evaluate the real‐world outcomes and progression patterns in patients with BRAF^V600^‐mutant advanced melanoma treated with first‐line BRAFi‐based TT alone or combined therapy with anti‐PD‐1 from three centers in China. The objective of this study aimed to provide evidence for optimizing first‐line treatment and useful indication for future prospective trials in China.

## 2 RESULTS

### 2.1 Baseline patient characteristics

**FIGURE 1:**
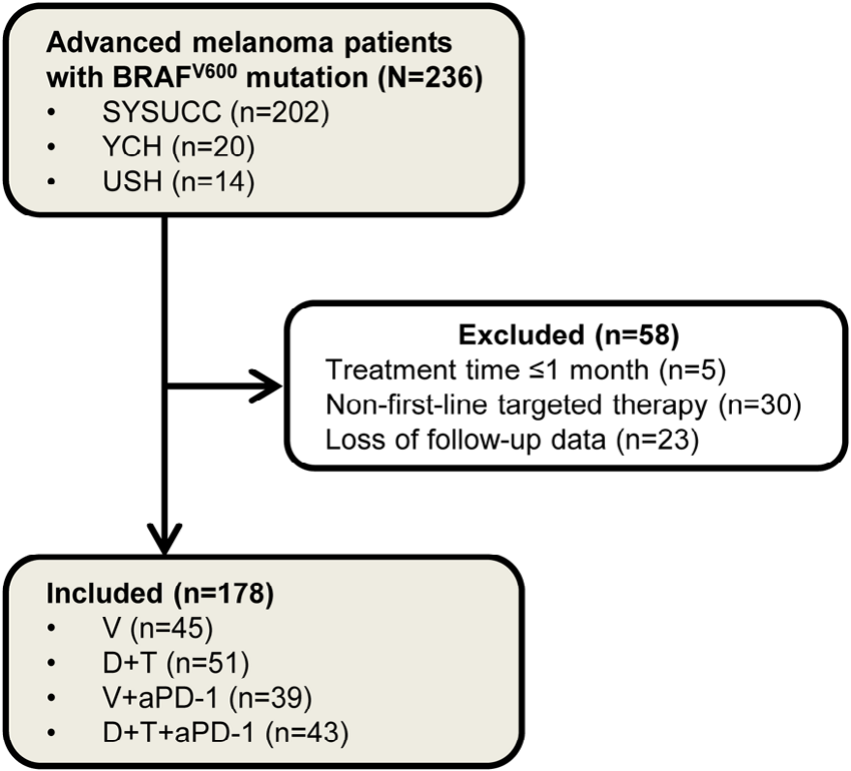
Flow diagram of patient screening. SYSUCC, Sun Yat‐sen University Cancer Center; YCH, Yunnan Cancer Hospital; USH, Union Shenzhen Hospital; V, vemurafenib; D, dabrafenib; T, trametinib; aPD‐1, anti‐PD‐1 antibody.

In the period between January 1, 2014 and May 1, 2023, a total of 236 patients with BRAF^V600^‐mutant advanced melanoma were screened from three centers in China. Following the exclusion of 58 patients who failed to meet the inclusion criteria, 178 patients (median age 49.5 years [IQR 38–57]; 76 [42.7%] men and 102 [57.3%] women) were included in the present study (Figure 1; Table 1). The included patients were divided into four groups: V (25.3%), D+T (28.7%), V+anti‐PD‐1 (21.9%) and D+T+anti‐PD‐1 (24.2%). The cutaneous, acral and mucosal subtypes accounted for 78.4%, 17.0% and 4.6%, respectively. BRAF^V600E^ mutation was detected in 115 (64.6%) patients, whereas BRAF^V600K^ was identified in only 4 (2.2%) patients. 59 (33.1%) patients were found to carry the BRAF^V600E/K^ mutation, highlighting the limitation of the Cobas® 4800 BRAF^V600^ Mutation Test in differentiating between V600E and V600K variants. No significant differences were observed in baseline characteristics among groups. Comprehensive details are provided in Table S1.

**TABLE 1:** Baseline characteristics of patients stratified by first‐line therapy.

	MAPKi		MAPKi + aPD‐1		
Characteristics	V (*n* = 45)	D+T (*n* = 51)	V+aPD‐1 (*n* = 39)	D+T+aPD‐1 (*n* = 43)	*p* value
**Age, median (range), years**	52 (27–75)	50 (24–80)	48 (24–71)	46 (21–78)	
**Age (*n*, %)**					0.530
<60	36 (80.0)	37 (72.5)	33 (84.6)	35 (81.4)	
≥60	9 (20.0)	14 (27.5)	6 (15.4)	8 (18.6)	
**Gender (*n*, %)**					0.451
Male	23 (51.1)	19 (37.3)	18 (46.2)	16 (37.2)	
Female	22 (48.9)	32 (62.7)	21 (53.8)	27 (62.8)	
**ECOG PS (*n*, %)**					0.259
0	36 (80.0)	43 (84.3)	31 (79.5)	40 (93.0)	
1–2	9 (20.0)	8 (15.7)	8 (20.5)	3 (7.0)	
**Serum LDH (*n*, %)**					0.111
Normal	24 (53.3)	33 (64.7)	33 (84.6)	30 (69.8)	
Elevated	16 (35.6)	12 (23.5)	4 (10.3)	9 (20.9)	
Missing	5 (11.1)	6 (11.8)	2 (5.1)	4 (9.3)	
**Subtype (*n*, %)**					0.188
Acral	5 (11.1)	11 (21.6)	9 (23.1)	3 (7.0)	
Mucosal	4 (8.9)	4 (7.8)	2 (5.1)	2 (4.7)	
CSD	5 (11.1)	11 (21.6)	10 (25.6)	8 (18.6)	
Non‐CSD	31 (68.9)	25 (49.0)	18 (46.2)	30 (69.8)	
**Tumor stage (*n*, %)**					0.273
Stage III	5 (11.1)	12 (23.5)	8 (20.5)	5 (11.6)	
Stage IV	40 (88.9)	39 (76.5)	31 (79.5)	38 (88.4)	
**Number of metastatic sites (*n*, %)**					0.152
<3	23 (51.1)	28 (54.9)	28 (71.8)	29 (67.4)	
≥3	22 (48.9)	23 (45.1)	11 (28.2)	14 (32.6)	
**Brain metastasis (*n*, %)**					0.382
No	34 (75.6)	37 (72.5)	34 (87.2)	32 (74.4)	
Yes	11 (24.4)	14 (27.5)	5 (12.8)	11 (25.6)	
**Liver metastasis (*n*, %)**					0.794
No	33 (73.3)	38 (74.5)	31 (79.5)	30 (69.8)	
Yes	12 (26.7)	13 (25.5)	8 (20.5)	13 (30.2)	
**Lung metastasis (*n*, %)**					0.603
No	18 (40.0)	24 (47.1)	20 (51.3)	23 (53.5)	
Yes	27 (60.0)	27 (52.9)	19 (48.7)	20 (46.5)	

Abbreviations: aPD‐1, anti‐PD‐1 antibody; CSD, chronic sun‐induced damage; D, dabrafenib; ECOG PS, Eastern Cooperative Oncology Group Performance Status; LDH, lactate dehydrogenases; MAPKi, inhibitor of mitogen‐activated protein kinases; T, trametinib; V, vemurafenib.

### 2.2 Treatment outcomes

At the data cut‐off for analysis (December 1, 2023), the median follow‐up time was 37.9 months (IQR 26.1–55.1) for the entire cohort. The follow‐up period differed among groups due to the timing of drug approval (Table S2). The overall response rate (ORR) of first‐line V, D+T, V+anti‐PD‐1, and D+T+anti‐PD‐1 were 46.7%, 54.9%, 51.3%, and 65.1%, respectively (*p* = 0.357) (Table S3). There was no significant difference in efficacy among groups (Table S4).

**FIGURE 2:**
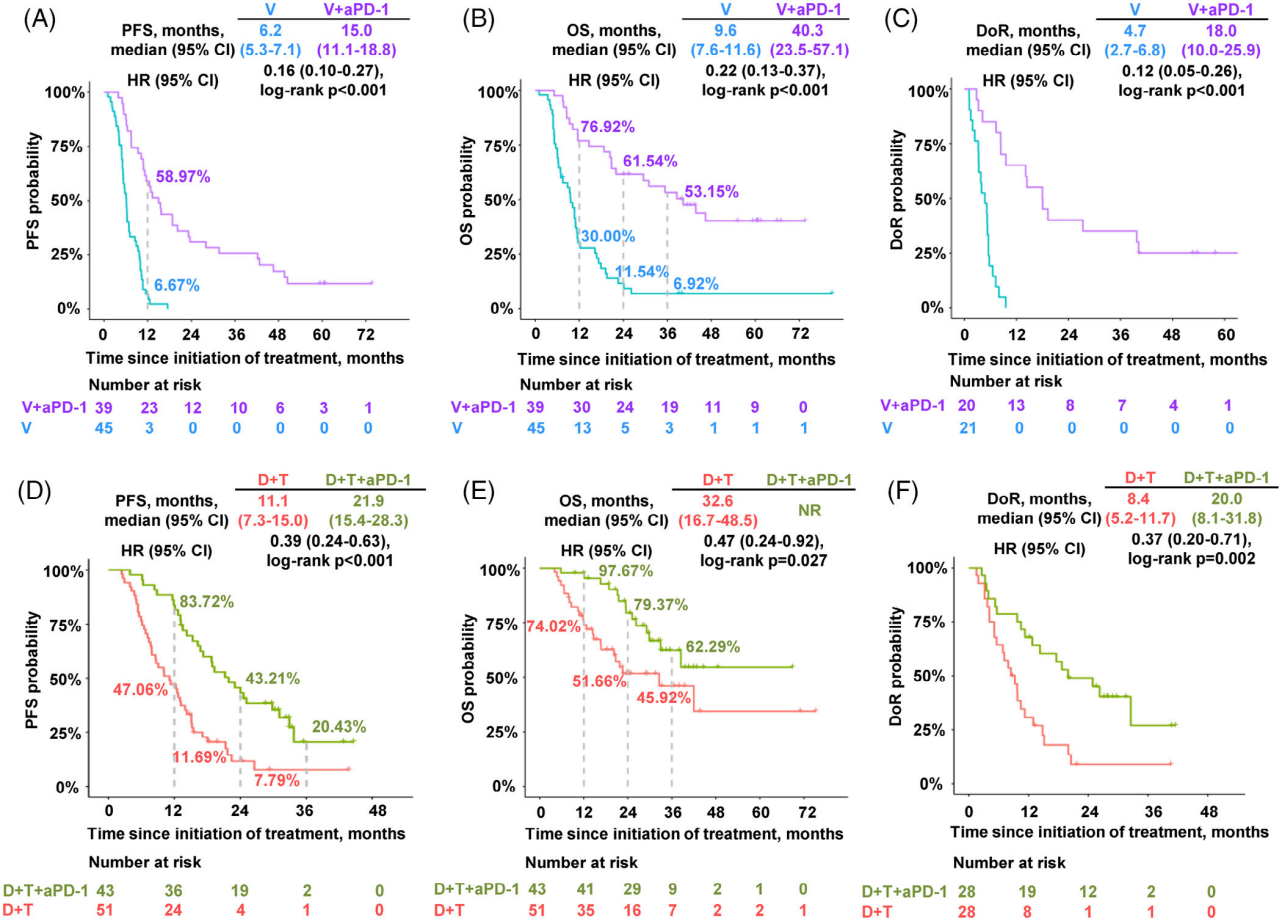
Addition of anti‐PD‐1 to BRAF inhibitor‐based targeted therapy improved survival. Kaplan–Meier curves showing (A) PFS, (B) OS, and (C) DoR for the V+aPD‐1 and V groups; (D) PFS, (E) OS, and (F) DoR for the D+T+aPD‐1 and D+T groups. NR, not reached. V, vemurafenib; D, dabrafenib; T, trametinib; aPD‐1, anti‐PD‐1 antibody; PFS, progression‐free survival; OS, overall survival; DoR, duration of response; HR, hazard ratio.

First of all, we compared the D+T and V groups to ascertain the effect of adding MEKi to BRAFi. D+T showed a significantly longer PFS, overall survival (OS), and duration of response (DoR) compared to V (*p* < 0.001) (Figure S1A–C). Next, we focused on the effect of adding anti‐PD‐1 to BRAFi‐based TT. We found that the addition of anti‐PD‐1 to V significantly prolonged PFS, OS, and DoR (*p* < 0.001) (Figure 2A–C). D+T+anti‐PD‐1 also showed a significantly longer PFS, OS, and DoR than D+T (PFS: 21.9 vs. 11.1 months, HR 0.39, *p* < 0.001; OS: NR vs. 32.6 months, HR 0.47, *p* = 0.027; DoR: 20.0 vs. 8.4 months, HR 0.37, *p* = 0.002) (Figure 2D–F).

**FIGURE 3:**
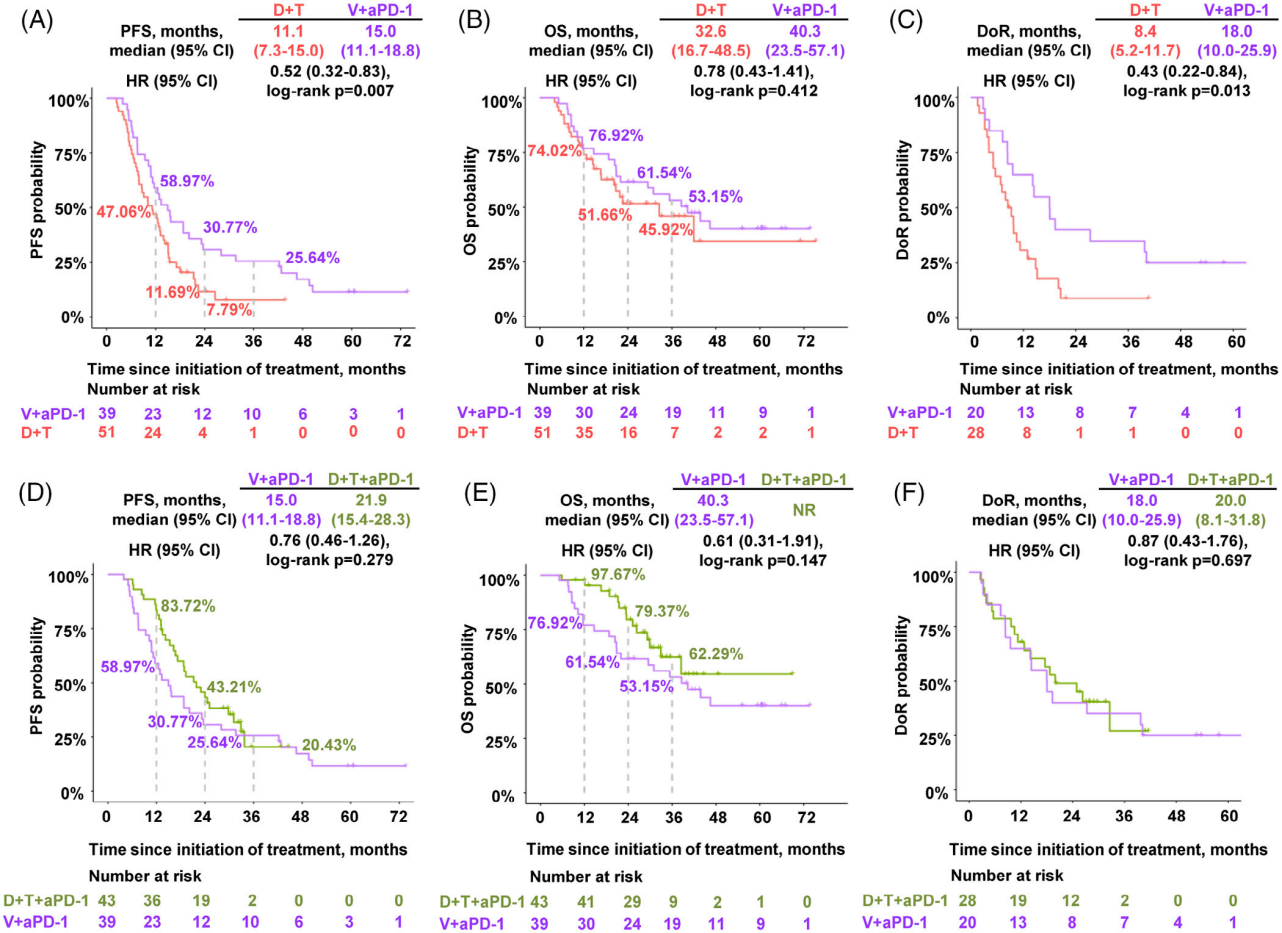
V+anti‐PD‐1 appeared to be an effective alternative treatment. Kaplan–Meier curves showing (A) PFS, (B) OS, and (C) DoR for the V+aPD‐1 and D+T groups; (D) PFS, (E) OS, and (F) DoR for the D+T+aPD‐1 and V+aPD‐1 groups. NR, not reached. V, vemurafenib; D, dabrafenib; T, trametinib; aPD‐1, anti‐PD‐1 antibody; PFS, progression‐free survival; OS, overall survival; DoR, duration of response; HR, hazard ratio.

In the comparison between V+anti‐PD‐1 and D+T, V+anti‐PD‐1 was superior to D+T in terms of PFS (15.0 vs. 11.1 months, HR 0.52, *p* = 0.007) and DoR (18.0 vs. 8.4 months, HR 0.43, *p* = 0.013), although no additional survival benefit was demonstrated (Figure 3A–C). We also found that neither was significantly different between V+anti‐PD‐1 and D+T+anti‐PD‐1 (Figure 3D–F), thus indicating that V+anti‐PD‐1 may be an effective alternative treatment option.

In addition, univariable and multivariable Cox regression analyses revealed that patients with Eastern Cooperative Oncology Group Performance Status (ECOG PS) 0 and normal serum lactate dehydrogenase (LDH) at baseline had a longer PFS and OS when treated with various first‐line treatment (Tables S5–S7).

### 2.3 Patterns of failure

**FIGURE 4:**
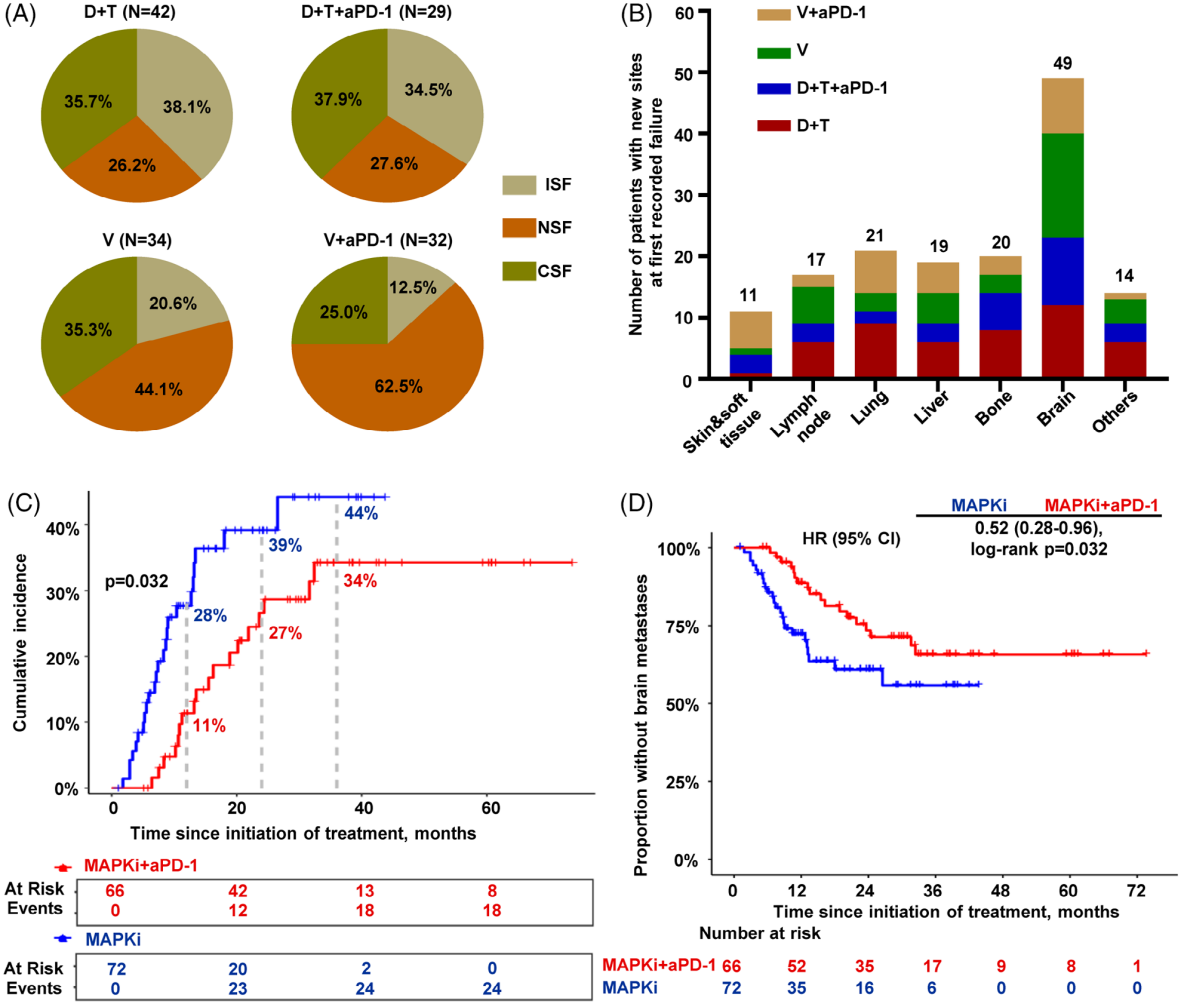
Addition of anti‐PD‐1 to BRAF inhibitor‐based targeted therapy delayed BM. (A) Pie chart showing the incidence of various patterns of failure in each group. (B) Bar chart showing the number of patients with new sites at first recorded failure in each group. (C) Cumulative incidence of brain metastases with MAPKi or MAPKi+aPD‐1. (D) Kaplan–Meier curves showing the time to development of BM with MAPKi or MAPKi+aPD‐1. BM, brain metastasis; MAPKi, inhibitor of mitogen‐activated protein kinases; V, vemurafenib; D, dabrafenib; T, trametinib; aPD‐1, anti‐PD‐1 antibody; ISF, initial‐site failure; NSF, new‐site failure; CSF, combined‐site failure; HR, hazard ratio.

At data cut‐off, 137 of the included patients demonstrated progression, with a median time to progression of 10.7 months (95% CI 9.3–12.1). Of these, 37 (27.0%) had ISF, 54 (39.4%) had NSF, and 46 (33.6%) had CSF. V and V+anti‐PD‐1 had similar patterns of failure, which mainly manifested as the emergence of new sites, whereas progression of the original disease was the main reason for treatment failure in D+T and D+T+anti‐PD‐1 (Figure 4A). The most common new metastatic sites at first recorded failure were brain (35.8%), lung (15.3%), bone (14.6%) and liver (13.9%) (Figure 4B). This suggests that patients are most likely to develop brain metastases (BM) at disease progression.

Due to the common emergence of BM, we analyzed the cumulative incidence of BM and time to development of BM in 138 patients with no history or evidence of BM at baseline. Based on the first‐line treatment regimens, the patients were categorized into two groups: the inhibitor of mitogen‐activated protein kinase (MAPKi) group, including patients treated with V or D+T (*n* = 72); and the MAPKi+anti‐PD‐1 group, including patients treated with V+anti‐PD‐1 or D+T+anti‐PD‐1 (*n* = 66). At the time of analysis, 27.3% (18/66) of patients in the MAPKi cohort and 33.3% (24/72) of patients in the MAPKi+anti‐PD‐1 cohort had developed BM. The cumulative incidence of BM at 1, 2 and 3 years in the MAPKi+aPD‐1 cohort (10%, 25% and 37%, respectively) was lower than that in the MAPKi cohort (25%, 40% and 47%, respectively) (Figure 4C). Furthermore, a prolonged delay in the development of BM was observed in the MAPKi+anti‐PD‐1 cohort in comparison with the MAPKi cohort (HR 0.52; *p* = 0.032) (Figure 4D).

### 2.4 Safety assessment

The incidence of any grade treatment‐related adverse events (trAEs) was 73.3%, 72.5%, 97.4% and 95.3% in the V, D+T, V+anti‐PD‐1 and D+T+anti‐PD‐1 groups, respectively. The incidence of grade ≥ 3 trAEs was 24.4%, 21.6%, 46.2% and 44.2%, respectively (Table S8). The most common grade ≥ 3 trAEs were rash (13.3%, 3.9%, 17.9% and 16.3% in the V, D+T, V+anti‐PD‐1 and D+T+anti‐PD‐1 groups, respectively), pyrexia (2.2%, 3.9%, 2.6% and 16.3%, respectively), and arthralgia (4.4%, 2.0%, 12.8% and 4.7%, respectively). Treatment was discontinued due to trAEs in several patients: 1 (2.2%) in the V group due to rash and photosensitivity reaction; 1 (2.0%) in the D+T group due to pyrexia and myalgia; 1 (2.6%) in the V+anti‐PD‐1 group due to pneumonia; and 3 (7.0%) in the D+T+anti‐PD‐1 group due to rash, pyrexia and uveitis (Table S9). The incidence of grade 3–5 immune‐mediated AEs was comparable between V+anti‐PD‐1 and D+T+anti‐PD‐1 groups (17.9% vs. 18.6%) (Table S10). Overall, the treatments were generally safe and well tolerated, with no deaths or other unexpected treatment‐related toxicities in the enrolled patients.

## 3 DISCUSSION

In this study, we analyzed the real‐world outcomes and progression patterns of first‐line V, D+T, V+anti‐PD‐1 and D+T+anti‐PD‐1 therapies in 178 Chinese patients from three centers. Our results suggest that the addition of anti‐PD‐1 to BRAFi‐based TT is a safe and effective strategy that delays resistance to TT and reduces the incidence of BM in patients with BRAF^V600^‐mutant advanced melanoma.

As found in our study, the addition of anti‐PD‐1 to D+T significantly prolonged PFS, OS, and DoR, with an increased but acceptable incidence of grade ≥3 trAEs and a low rate of treatment discontinuation. IMspire150 study also showed that triple therapy had an additional benefit over TT alone and was the only trial to show a statistically significant PFS.^22^, ^23^ It is worthy of note that, in comparison to the data from prospective trials such as KEYNOTE‐022, IMspire150, and COMBI‐i, patients in our study who received triplet therapy appeared to experience superior treatment outcomes, with a longer median PFS (21 months vs. 15–18 months). This may be attributed to the lower baseline tumor burden of the enrolled patients in this study, as evidenced by significantly reduced proportions of patients with ≥3 metastatic sites, stage IV, M1c, and elevated LDH. Additionally, it is crucial that patients in our study benefited from the run‐in period of TT, demonstrating enhanced tolerability with lower rates of trAEs and treatment discontinuation. A run‐in period of TT prior to additional intravenous anti‐PD‐1 was carried out in our study and IMspire 150, which differed from KEYNOTE‐022^24^, ^25^ and COMBI‐i study^26^ that used three‐drug combinations at the same time. The incidence of grade ≥3 trAEs in patients undergoing triplet therapy in our study was 46.2%, much lower than the 70% reported in the KEYNOTE‐022 study, and the incidence of treatment discontinuation was also much lower in our study (5.1% vs. 47%). The optimized combination strategy including a run‐in period of TT could not only induce a more favorable tumor immune microenvironment, but also shrink tumors, promote tumor antigen release and enhance immunogenicity especially for Chinese melanoma patients characterized by heavy tumor load and late stage at diagnosis.^30^ Therefore, introducing a run‐in period of TT before combination therapy is a recommended strategy to maximize clinical benefit, but more perspective trials are needed to find out the optimal combination regimen, sequence or timing of initiation of therapy.

Notably, we focused on the efficacy of V+anti‐PD‐1, a unique therapeutic modality that was widely used in our center especially prior to the approval of D+T in China. Currently, V monotherapy is no longer recommended abroad, but rather in combination with cobimetinib. However, cobimetinib is not yet available in China; both V monotherapy and anti‐PD‐1 are still first‐line treatment options for Chinese patients with BRAF^V600^‐mutant advanced melanoma. According to our study, V+anti‐PD‐1 showed a significantly longer PFS and DoR than D+T and was comparable to D+T+anti‐PD‐1. Although the incidence of grade ≥ 3 trAEs in the V+anti‐PD‐1 group is higher than that in the D+T group, it is still safe and controllable, and the toxicity profiles of them are different. Overall, our study offers a novel and effective alternative treatment, V+anti‐PD‐1, especially for those who cannot tolerate the side effects of D+T such as severe pyrexia or ocular toxicity. The findings of the research indicated that the prognosis outcomes for V+anti‐PD‐1 and D+T+anti‐PD‐1 were similar, with no statistically significant differences observed between the two groups. Although D+T+anti‐PD‐1 appeared to demonstrate a trend of superior in comparison to V+anti‐PD‐1, the limited sample size precludes any definitive conclusions. Further research could expand the sample size and conduct longer follow‐up periods in order to further verify the results regarding PFS and DoR for both groups with greater precision.

The progression patterns were analyzed after first‐line treatment in our study, which has rarely been reported in previous studies. Our preliminary findings indicated that progression in the V and V+anti‐PD‐1 groups primarily occurred at new sites, whereas the D+T and D+T+anti‐PD‐1 groups exhibited predominant progression at the initial sites. This observation suggests a potential correlation between progression sites and specific types of targeted therapies. However, the limitations in sample size significantly hinder our ability to draw definitive conclusions, highlighting the necessity for further research in this field to enhance our understanding and inform future developments. In addition, we found that brain was the most common new site at the first recorded failure across all treatment groups. Previous studies have shown that clinically acquired resistance to BRAFi/MEKi preferentially occurs in the brain and usually precedes extracranial disease progression.^31^, ^32^ In our clinical practice, patients often experience treatment failure due to the development of BM despite well‐controlled extracranial disease. The exploratory analyses from IMspire150 study also demonstrated a reduction in the risk of developing BM with triple therapy.^33^ Our study provides supportive evidence for the necessity of adding anti‐PD‐1 to TT in delaying BM, which is instructive to guide clinical decision making, but the specific mechanism still needs to be further investigated.

The limitations of the study were the relatively small sample size and the retrospective nature of the investigation; therefore, the study was susceptible to both recall and selection bias despite incredible efforts to mitigate potential biases. To the best of our knowledge, this study represents the first and largest retrospective multicenter study to investigate the real‐world outcomes and progression patterns of first‐line BRAFi‐based TT combined with anti‐PD‐1 in Asian patients. In addition, due to the lack of an indication for anti‐CTLA‐4 drugs in melanoma in China, along with other objective factors, there is a shortage of advanced melanoma patients undergoing combined immunotherapy in our study. Consequently, future studies should encompass patients treated with PD‐1/CTLA‐4 inhibitors, either alone or in combination, to facilitate more comprehensive comparative analyses.

## 4 CONCLUSION

In summary, the findings of this study suggest that the addition of anti‐PD‐1 to BRAFi‐based TT appears to be a safe and effective treatment option that provides a survival benefit and a delayed BM in BRAF^V600^‐mutant advanced melanoma patients in real‐world practice. Overall, this study provides supportive evidence for the first‐line combination therapies of anti‐PD‐1 and BRAFi‐based TT and confirms the value of adding anti‐PD‐1 to TT in delaying BM. It has been demonstrated that a combination strategy which incorporates a run‐in period of targeted therapy is optimized, and this combination strategy should be advocated in clinical practice. However, there are still many unanswered questions regarding the choice of drugs for combination therapy, the optimal sequence or timing of initiation of therapy and the best way to mitigate drug toxicity.

## 5 MATERIALS AND METHODS

### 5.1 Patients

From January 1, 2014 to May 1, 2023, eligible patients with BRAF^V600^‐mutant advanced melanoma were enrolled in this study from three medical institutions in China: Sun Yat‐sen University Cancer Center, Yunnan Cancer Hospital and Huazhong University of Science and Technology Union Shenzhen Hospital. The inclusion criteria were listed as follows: (1) pathologically confirmed melanoma; (2) BRAF^V600^‐mutant status; (3) unresectable stage III/IV melanoma; (4) received first‐line BRAFi‐based TT alone or in combination therapy with anti‐PD‐1. Patients with treatment time ≤1 month, or receiving other first‐line treatment, or with loss of follow‐up data were excluded (Figure 1).

Tumor stage was determined according to the 8th edition of the American Joint Committee on Cancer.^34^ Patient clinicopathological characteristics and follow‐up data were obtained from their medical records. The presence of BRAF mutations was detected by polymerase chain reaction or next‐generation sequencing of formalin‐fixed paraffin‐embedded tissue. Permission to conduct this study and a waiver for informed consent were obtained from the Ethics Committee of Sun Yat‐sen University Cancer Center (B2023‐586‐01). This study was performed in accordance with the Declaration of Helsinki.

### 5.2 Study treatment

Patients enrolled in the study were categorized into the following groups based on treatment regimen: V, D+T, V+anti‐PD‐1 and D+T+anti‐PD‐1. Patients in the V group received twice‐daily oral vemurafenib 960 mg, whereas patients in the D+T group received twice‐daily oral dabrafenib 150 mg and once‐daily oral trametinib 2 mg. Patients in the V+anti‐PD‐1 or D+T+anti‐PD‐1 group received 4–6 weeks of V or D+T run‐in followed by concurrent intravenous anti‐PD‐1 (pembrolizumab 200 mg or toripalimab 240 mg every 3 weeks) plus V or D+T. Toripalimab is another anti‐PD‐1 that developed by domestic pharmaceutical company and approved for melanoma.^35^, ^36^ The duration of the treatment is calculated from the commencement of the run‐in period for TT. Patients who exhibited rapid progression despite initial anti‐PD‐1 were included in the analysis.

Patients were all scheduled to receive first‐line therapy until disease progression or for a maximum of 2 years. Decisions regarding dose reduction, dose interruption or treatment discontinuation were made considering the management of trAEs and treatment tolerance of patients.

### 5.3 Efficacy and prognosis

Tumor response was determined using Response Evaluation Criteria in Solid Tumours (RECIST) version 1.1.^37^ The following endpoints were evaluated: ORR, the proportion of patients with a partial or complete response; DoR, the time from the first partial or complete response to disease progression, death or last follow‐up; PFS, the time from therapy initiation to disease progression, death or last follow‐up; and OS, the time from therapy initiation to death or the last follow‐up.

### 5.4 Patterns of failure

Follow‐up radiological scans were evaluated by two independent investigators to identify progression of the original disease (primary/metastatic sites) or new sites of disease. The patterns of first recorded failure were characterized as follows: initial‐site failure (ISF), the first failure with evidence of progression of primary or metastatic sites that were present before treatment; new‐site failure (NSF), the first failure with the radiographic emergence of new sites that were uninvolved at baseline; and combined‐site failure (CSF), the first failure with evidence of ISF and NSF detected simultaneously. The new sites and the date of first documented failure were also recorded. Cumulative incidence of BM and the time to development of BM were performed in patients with no history or evidence of BM at baseline.

### 5.5 Safety assessment

The severity of trAEs was graded and recorded according to the National Cancer Institute Common Terminology Criteria for Adverse Events version 4.0.

### 5.6 Statistics

The correlation analyses were performed using the *χ* ^2^ test and Fisher's exact tests, whereas survival analyses were performed by the Kaplan–Meier method. A Cox proportional hazard model was used for univariate and multivariate analyses. Cumulative incidence of BM was evaluated using competing risk analysis and the time to the development of BM was estimated using the Kaplan–Meier method with adjustment for death and other types of progressive disease. All statistical analyses were performed with IBM SPSS version 22.0 software and R version 4.2.0 software. A *p*‐value of < 0.05 was considered statistically significant.

## AUTHOR CONTRIBUTIONS

The study conception and design were collaboratively developed by Dandan Li, Xiaoshi Zhang, Ke Li, Qiming Zhou, and Junwan Wu. Data acquisition was carried out by Junwan Wu, Qiuyue Ding, Qiong Zhang, Qianqi Chen, Xizhi Wen, Ziluan Chen, Tao Zhang, Fuxue Huang, Linbin Chen, and Ke Li. Junwan Wu extracted the data and performed statistical analysis under the supervision and guidance of Jiuhong Wang and Hang Jiang. Dandan Li, Xiaoshi Zhang, Ke Li, Qiming Zhou, Xizhi Wen, Ya Ding, and Jingjing Li accessed and validated the data. Junwan Wu completed the writing of the initial draft. Dandan Li, Qiuyue Ding, Qiong Zhang, and Jingjing Li revised and polished the manuscript. All authors contributed to data interpretation, writing, and reviewing the manuscript, being fully accountable for the data integrity and the decision to publish. All authors have read and approved the final manuscript.

## CONFLICT OF INTEREST STATEMENT

All authors declare no financial or nonfinancial competing interests.

## ETHICS STATEMENT

This study was conducted with the approval of the Ethics Committee of Sun Yat‐sen University Cancer Center (B2023‐586‐01) and performed in accordance with the Declaration of Helsinki. A waiver for informed consent was obtained.

## Supporting information



Supporting information

## Data Availability

Due to patient privacy issues, relevant data cannot be made available to the public. However, it can be obtained upon reasonable request from Dandan Li (lidd@sysucc.org.cn). The principal raw data has also been uploaded to the Research Data Deposit public platform (www.researchdata.org.cn), with the approval number of RDDA2025396124.
