# Supplementary material for: Addition of anti‐PD‐1 immunotherapy to BRAF inhibitor‐based targeted therapy improves real‐world survival and delays brain metastases in patients with BRAFV600‐mutant advanced melanoma: a multicenter cohort study
Source: MedComm (2020). 2025 Feb 17;6(3):e70102. doi: 10.1002/mco2.70102 (PMC11832434; doi:10.1002/mco2.70102)
Supplement: Supplementary file 1 — Supporting information [file MCO2-6-e70102-s001.docx]

**Supplementary Materials**

Addition of anti-PD-1 immunotherapy to BRAF inhibitor-based targeted therapy improves real-world survival and delays brain metastases in patients with BRAF^V600^-mutant advanced melanoma: a multicenter cohort study

Junwan Wu^1,2#^, Qiuyue Ding^1,2#^, Qiong Zhang^1,2#^, Qianqi Chen^3,#^, Xizhi Wen^1,2^, Ya Ding^1,2^, Jingjing Li^1,2^, Ziluan Chen^1,2^, Tao Zhang^2^, Jiuhong Wang^1,2^, Fuxue Huang^1,2^, Hang Jiang^1,2^, Linbin Chen^1,2^, Qiming Zhou^3,*^, Ke Li^4,*^, Xiaoshi Zhang^1,2,*^, Dandan Li^1,2,*^

^1^Biotherapy Center, Sun Yat-sen University Cancer Center, State Key Laboratory of Oncology in South China, Guangdong Provincial Clinical Research Center for Cancer, Collaborative Innovation Center for Cancer Medicine, Guangzhou 510060, P. R. China.

^2^State Key Laboratory of Oncology in South China, Guangdong Provincial Clinical Research Center for Cancer, Collaborative Innovation Center for Cancer Medicine, Sun Yat-sen University Cancer Center, Guangzhou 510060, P. R. China.

^3^Department of Oncology, Huazhong University of Science and Technology Union Shenzhen Hospital, Shenzhen 518052, P. R. China.

^4^Department of Cancer Biotherapy Center, Yunnan Cancer Hospital, The Third Affiliated Hospital of Kunming Medical University, Cancer Center of Yunnan Province, Kunming 650118, P. R. China.

^#^Junwan Wu, Qiuyue Ding, Qiong Zhang and Qianqi Chen contributed equally.

**^*^Correspondence**

Dandan Li and Xiaoshi Zhang, Biotherapy Center,

Sun Yat-sen University Cancer Center, Guangzhou, China.

Email: [lidd@sysucc.org.cn](mailto:lidd@sysucc.org.cn); zhangxsh@sysucc.org.cn

Ke Li, Department of Cancer Biotherapy Center,

Yunnan Cancer Hospital, Yunnan, China.

Email: [likelikelike@126.com](mailto:likelikelike@126.comshihb@scu.edu.cn)

Qiming Zhou, Department of Oncology,

Huazhong University of Science and Technology Union Shenzhen Hospital, Shenzhen, China.

E-mail: [zqm961221@163.com](mailto:zqm961221@163.com)

**Supplementary Materials**

**Figure S1.** D+T was superior to V in prolonging survival.

**Table S1.** Patient demographics, clinical features and survival outcomes.

**Table S2.** Follow-up time of patients stratified by first-line therapy.

**Table S3.** Treatment response of patients stratified by first-line therapy.

**Table S4.** Intergroup comparison of treatment response.

**Table S5.** Univariable analysis of prognostic factors correlated with PFS.

**Table S6.** Univariable analysis of prognostic factors correlated with OS.

**Table S7.** Multivariable analyses of prognostic factors correlated with PFS and OS.

**Table S8.** Treatment-related adverse events of patients stratified by first-line therapy.

**Table S9.** Adverse events leading to dose reduction, dose interruption and treatment discontinuation.

**Table S10.** Immune-mediated adverse events occurring in at least one patient in either treatment arm.


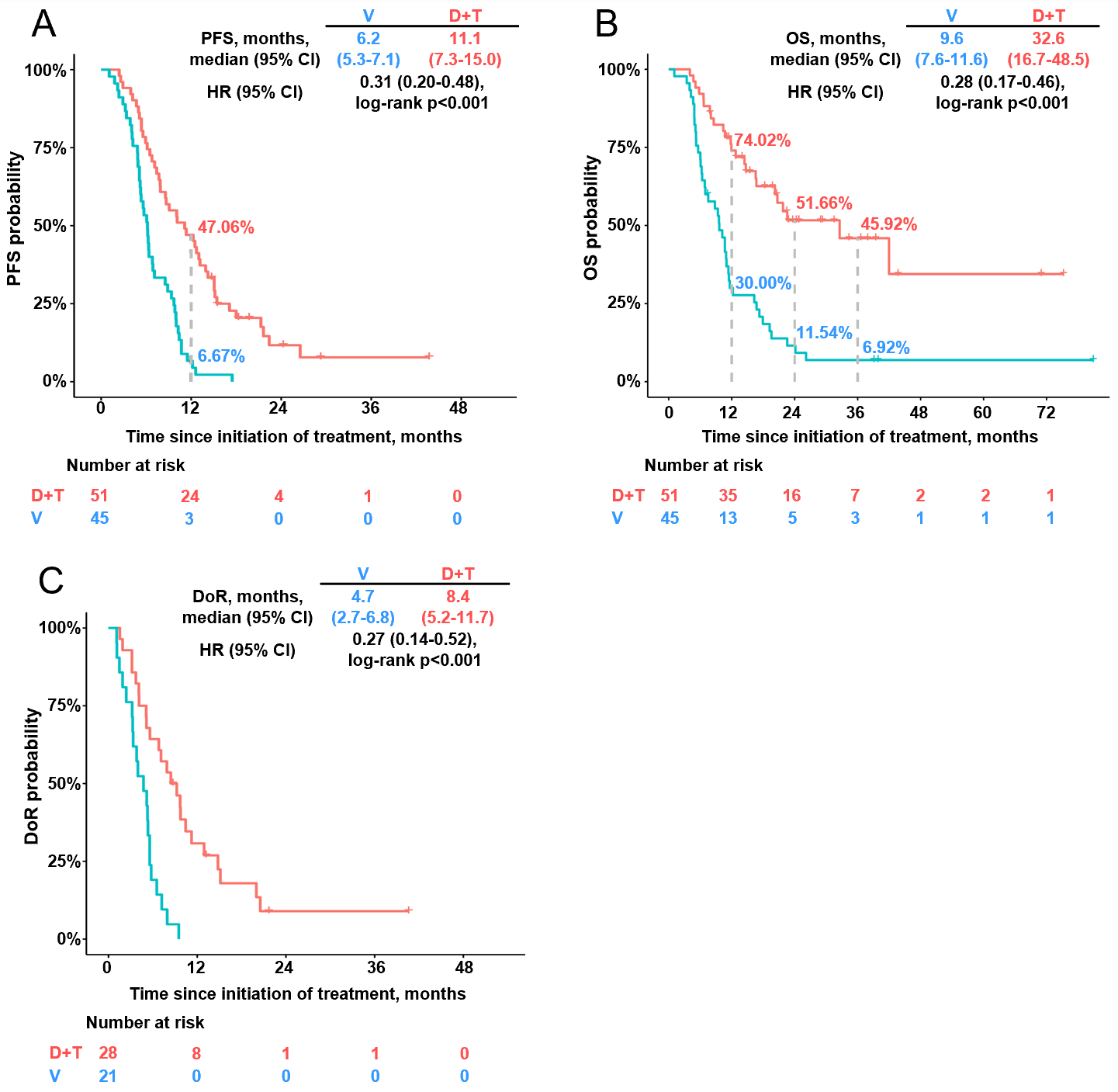


**Figure S1.** **D+T was superior to V in prolonging survival.** Kaplan‒Meier curves showing (A) PFS, (B) OS and (C) DoR for the V and D+T groups. V, vemurafenib; D, dabrafenib; T, trametinib; PFS, progression-free survival; OS, overall survival; DoR, duration of response; HR, hazard ratio.

**Table S1. Patient demographics, clinical features and survival outcomes.**

| **ID** | **Patients source** | **Age at therapy initiation** | **Gender** | **ECOG PS** | **Serum LDH** | **BRAF status** | **Subtype** | **Tumor stage** | **Number of metastatic sites** | **M stage** | **Brain metastasis at therapy initiation** | **Liver metastasis at therapy initiation** | **Lung metastasis at therapy initiation** | **Treatment** | **Efficacy** | **Patterns of failure** | **Progression status** | **Death status** | **Date of initiation** | **Date of first CR/PR** | **Date of progression/ last follow-up** | **Date of death/ last follow-up** | **Duration of response (months)** | **Progression free survival (months)** | **Overall survival (months)** | **Brain metastasis status** | **Date of brain metastasis /death/ last follow-up** | **Time to development of brain metastasis (months)** |
| --- | --- | --- | --- | --- | --- | --- | --- | --- | --- | --- | --- | --- | --- | --- | --- | --- | --- | --- | --- | --- | --- | --- | --- | --- | --- | --- | --- | --- |
| MELA 01 | SYSUCC | 42 | Male | 0 | Normal | V600E | Acral | IV | ＜3 | M1b | No | No | Yes | D+T+aPD-1 | PR | No progression | No | No | 2021/1/1 | 2021/4/14 | 2023/12/1 | 2023/12/1 | 32.03 | 35.47 | 35.47 | No | 2023/12/1 | 35.47 |
| MELA 02 | SYSUCC | 57 | Male | 0 | Normal | V600E | Non-CSD | IV | ＜3 | M1b | No | No | Yes | D+T+aPD-1 | PR | ISF | Yes | No | 2022/3/22 | 2022/5/27 | 2023/3/14 | 2023/12/1 | 9.70 | 11.90 | 20.63 | No | 2023/12/1 | 20.63 |
| MELA 03 | SYSUCC | 54 | Female | 1 | Elevated | V600E | Non-CSD | IV | ≥3 | M1c | No | Yes | Yes | V | SD | Death without failure | Yes | Yes | 2017/10/18 | / | 2018/3/14 | 2018/3/14 | / | 4.90 | 4.90 | No | 2018/3/14 | 4.90 |
| MELA 04 | SYSUCC | 67 | Male | 0 | Normal | V600E | Non-CSD | IV | ≥3 | M1d | Yes | Yes | Yes | D+T | SD | Death without failure | Yes | Yes | 2021/3/8 | / | 2021/11/20 | 2021/11/20 | / | 8.57 | 8.57 | Yes (at therapy initiation) | / | / |
| MELA 05 | SYSUCC | 66 | Male | 0 | Normal | V600E | Non-CSD | IV | ＜3 | M1a | No | No | No | D+T+aPD-1 | PR | NSF | Yes | No | 2021/10/9 | 2021/12/5 | 2022/11/8 | 2023/12/1 | 11.27 | 13.17 | 26.10 | Yes (within or after treatment) | 2022/11/8 | 13.17 |
| MELA 06 | SYSUCC | 50 | Female | 0 | Normal | V600E/K | Non-CSD | IV | ＜3 | M1b | No | No | Yes | V+aPD-1 | PR | ISF | Yes | Yes | 2018/5/1 | 2018/6/16 | 2019/8/14 | 2021/7/2 | 14.13 | 15.67 | 38.60 | No | 2021/7/2 | 38.60 |
| MELA 07 | SYSUCC | 37 | Male | 1 | Elevated | V600E/K | Non-CSD | IV | ≥3 | M1d | Yes | No | Yes | V | SD | Death without failure | Yes | Yes | 2019/5/28 | / | 2019/10/31 | 2019/10/31 | / | 5.20 | 5.20 | Yes (at therapy initiation) | / | / |
| MELA 08 | SYSUCC | 41 | Female | 0 | Normal | V600E | CSD | IV | ≥3 | M1c | No | Yes | Yes | V+aPD-1 | SD | CSF | Yes | No | 2020/8/19 | / | 2021/6/22 | 2022/8/11 | / | 10.23 | 24.07 | Yes (within or after treatment) | 2021/6/22 | 10.23 |
| MELA 09 | SYSUCC | 63 | Female | 0 | Normal | V600E/K | Acral | IV | ≥3 | M1b | No | No | Yes | D+T | PR | NSF | Yes | No | 2017/9/26 | 2018/12/29 | 2019/12/1 | 2023/12/1 | 11.23 | 26.53 | 75.23 | Yes (within or after treatment) | 2019-12-1 | 26.53 |
| MELA 10 | SYSUCC | 35 | Male | 1 | Elevated | V600E | CSD | IV | ≥3 | M1d | Yes | Yes | Yes | D+T+aPD-1 | PR | No progression | No | No | 2021/3/22 | 2021/5/19 | 2023/12/1 | 2023/12/1 | 30.87 | 32.80 | 32.80 | Yes (at therapy initiation) | / | / |
| MELA 11 | SYSUCC | 59 | Male | 0 | Normal | V600E/K | Non-CSD | IIIC | ＜3 | M0 | No | No | No | V+aPD-1 | PR | NSF | Yes | No | 2018/6/19 | 2018/10/15 | 2020/5/15 | 2020/7/22 | 19.27 | 23.20 | 25.47 | Yes (within or after treatment) | 2020/5/27 | 23.60 |
| MELA 12 | SYSUCC | 57 | Female | 0 | Normal | V600E | CSD | IV | ＜3 | M1b | No | No | Yes | D+T | CR | No progression | No | No | 2021/12/3 | 2022/2/19 | 2023/12/1 | 2023/12/1 | 21.67 | 24.27 | 24.27 | No | 2023/12/1 | 24.27 |
| MELA 13 | SYSUCC | 28 | Male | 0 | Normal | V600E/K | Non-CSD | IV | ＜3 | M1d | Yes | No | No | V | SD | ISF | Yes | Yes | 2017/6/18 | / | 2017/12/6 | 2017/12/26 | / | 5.70 | 6.37 | Yes (at therapy initiation) | / | / |
| MELA 14 | SYSUCC | 48 | Male | 0 | Elevated | V600E/K | Non-CSD | IV | ≥3 | M1d | Yes | Yes | Yes | V | SD | Death without failure | Yes | Yes | 2019/7/1 | / | 2019/12/1 | 2019/12/1 | / | 5.10 | 5.10 | Yes (at therapy initiation) | / | / |
| MELA 15 | SYSUCC | 53 | Female | 0 | Elevated | V600E | Non-CSD | IV | ≥3 | M1c | No | No | Yes | D+T | PD | NSF | Yes | Yes | 2021/12/30 | / | 2022/3/27 | 2023/5/15 | / | 2.90 | 16.70 | Yes (within or after treatment) | 2022-3-27 | 2.90 |
| MELA 16 | SYSUCC | 54 | Female | 1 | Normal | V600E/K | Acral | IV | ≥3 | M1d | Yes | No | Yes | V | SD | ISF | Yes | Yes | 2019/8/14 | / | 2020/4/26 | 2020/12/15 | / | 8.53 | 16.30 | Yes (at therapy initiation) | / | / |
| MELA 17 | SYSUCC | 58 | Female | 0 | Normal | V600E | CSD | IV | ＜3 | M1c | No | No | Yes | V+aPD-1 | SD | NSF | Yes | No | 2020/5/8 | / | 2021/3/29 | 2023/12/1 | / | 10.83 | 43.40 | Yes (within or after treatment) | 2021/3/29 | 10.83 |
| MELA 18 | SYSUCC | 37 | Female | 1 | Elevated | V600E/K | Acral | IV | ≥3 | M1c | No | Yes | Yes | V+aPD-1 | SD | ISF | Yes | Yes | 2018/12/7 | / | 2019/4/4 | 2019/5/10 | / | 3.93 | 5.13 | No | 2019/5/10 | 5.13 |
| MELA 19 | SYSUCC | 50 | Female | 0 | Normal | V600E/K | Non-CSD | IV | ＜3 | M1b | No | No | Yes | V+aPD-1 | SD | NSF | Yes | Yes | 2019/11/25 | / | 2020/10/27 | 2023/3/18 | / | 11.23 | 40.30 | Yes (within or after treatment) | 2020/10/27 | 11.23 |
| MELA 20 | SYSUCC | 55 | Male | 0 | Normal | V600E | Non-CSD | IV | ＜3 | M1b | No | No | Yes | V | PR | NSF | Yes | Yes | 2018/11/21 | 2019/4/11 | 2019/9/16 | 2020/9/28 | 5.27 | 9.97 | 22.57 | No | 2020/9/28 | 22.57 |
| MELA 21 | SYSUCC | 70 | Male | 0 | Normal | V600E | Non-CSD | IV | ≥3 | M1d | Yes | No | Yes | D+T+aPD-1 | PR | No progression | No | No | 2021/6/23 | 2021/8/20 | 2023/12/1 | 2023/12/1 | 27.77 | 29.70 | 29.70 | Yes (at therapy initiation) | / | / |
| MELA 22 | SYSUCC | 36 | Female | 0 | Missing | V600E | Non-CSD | IV | ≥3 | M1c | No | Yes | Yes | V+aPD-1 | SD | NSF | Yes | Yes | 2019/6/17 | / | 2020/12/31 | 2022/1/1 | / | 18.77 | 30.97 | No | 2022/1/1 | 30.97 |
| MELA 23 | SYSUCC | 41 | Female | 0 | Missing | V600E | Non-CSD | IV | ≥3 | M1c | No | Yes | Yes | V | SD | Death without failure | Yes | Yes | 2019/6/13 | / | 2019/12/18 | 2019/12/18 | / | 6.27 | 6.27 | No | 2019/12/18 | 6.27 |
| MELA 24 | SYSUCC | 54 | Male | 0 | Normal | V600E | Non-CSD | IV | ≥3 | M1c | No | Yes | Yes | V | PR | CSF | Yes | Yes | 2019/1/15 | 2019/3/13 | 2019/4/26 | 2019/6/4 | 1.47 | 3.37 | 4.67 | Yes (within or after treatment) | 2019/4/26 | 3.37 |
| MELA 25 | SYSUCC | 54 | Male | 0 | Normal | V600E | Non-CSD | IIID | ＜3 | M0 | No | No | No | V+aPD-1 | PR | No progression | No | No | 2017/11/13 | 2018/6/13 | 2023/12/1 | 2023/12/1 | 66.57 | 73.63 | 73.63 | No | 2023/12/1 | 73.63 |
| MELA 26 | SYSUCC | 60 | Female | 1 | Elevated | V600E | Non-CSD | IV | ＜3 | M1b | No | No | Yes | V | PR | NSF | Yes | Yes | 2018/1/18 | 2018/3/12 | 2018/4/15 | 2018/5/1 | 1.13 | 2.90 | 3.43 | Yes (within or after treatment) | 2018-4-15 | 2.90 |
| MELA 27 | SYSUCC | 56 | Female | 0 | Normal | V600E/K | Non-CSD | IV | ＜3 | M1b | No | No | Yes | V | SD | Death without failure | Yes | Yes | 2018/12/7 | / | 2019/10/24 | 2019/10/24 | / | 10.70 | 10.70 | No | 2019/10/24 | 10.70 |
| MELA 28 | SYSUCC | 51 | Female | 0 | Normal | V600E | Acral | IV | ＜3 | M1b | No | No | Yes | V | PR | NSF | Yes | Yes | 2020/6/3 | 2020/8/21 | 2021/3/25 | 2022/1/11 | 7.20 | 9.83 | 19.57 | Yes (within or after treatment) | 2021-7-9 | 13.37 |
| MELA 29 | SYSUCC | 45 | Female | 1 | Elevated | V600K | CSD | IV | ≥3 | M1d | Yes | Yes | Yes | D+T+aPD-1 | SD | ISF | Yes | Yes | 2019/4/9 | / | 2020/4/23 | 2021/1/4 | / | 12.67 | 21.20 | Yes (at therapy initiation) | / | / |
| MELA 30 | SYSUCC | 61 | Female | 0 | Missing | V600E/K | Non-CSD | IV | ＜3 | M1c | No | No | No | V | PD | NSF | Yes | Yes | 2020/5/7 | / | 2020/6/30 | 2020/9/3 | / | 1.80 | 3.97 | Yes (within or after treatment) | 2020/6/30 | 1.80 |
| MELA 31 | SYSUCC | 71 | Male | 1 | Missing | V600K | Non-CSD | IV | ＜3 | M1c | No | Yes | No | D+T | SD | No progression | No | No | 2022/6/1 | / | 2023/12/1 | 2023/12/1 | / | 18.27 | 18.27 | No | 2023/12/1 | 18.27 |
| MELA 32 | SYSUCC | 50 | Female | 0 | Normal | V600K | CSD | IV | ≥3 | M1c | No | No | No | V | PR | CSF | Yes | Yes | 2019/10/18 | 2019/11/26 | 2020/3/24 | 2020/10/1 | 3.97 | 5.27 | 11.63 | Yes (within or after treatment) | 2020-3-24 | 5.27 |
| MELA 33 | SYSUCC | 71 | Female | 0 | Normal | V600E | Acral | IV | ＜3 | M1a | No | No | No | V+aPD-1 | SD | NSF | Yes | Yes | 2020/6/24 | / | 2021/2/3 | 2021/3/8 | / | 7.47 | 8.57 | No | 2021/3/8 | 8.57 |
| MELA 34 | SYSUCC | 52 | Male | 1 | Normal | V600K | CSD | IV | ≥3 | M1d | Yes | Yes | No | D+T | SD | ISF | Yes | Yes | 2020/11/13 | / | 2021/1/24 | 2021/5/6 | / | 2.40 | 5.80 | Yes (at therapy initiation) | / | / |
| MELA 35 | SYSUCC | 30 | Female | 0 | Normal | V600E | Non-CSD | IIIC | ＜3 | M0 | No | No | No | D+T | PR | NSF | Yes | Yes | 2021/6/18 | 2021/12/17 | 2022/7/18 | 2023/2/15 | 7.10 | 13.17 | 20.23 | Yes (within or after treatment) | 2022-7-18 | 13.17 |
| MELA 36 | SYSUCC | 33 | Male | 0 | Normal | V600E/K | CSD | IV | ＜3 | M1a | No | No | No | V+aPD-1 | PR | NSF | Yes | No | 2020/7/7 | 2020/9/9 | 2022/3/4 | 2023/12/1 | 18.03 | 20.17 | 41.40 | Yes (within or after treatment) | 2022/3/4 | 20.17 |
| MELA 37 | SYSUCC | 43 | Female | 0 | Normal | V600E | Non-CSD | IV | ＜3 | M1a | No | No | No | D+T+aPD-1 | PR | No progression | No | No | 2021/7/28 | 2021/10/23 | 2023/12/1 | 2023/12/1 | 25.63 | 28.53 | 28.53 | No | 2023/12/1 | 28.53 |
| MELA 38 | SYSUCC | 69 | Female | 0 | Normal | V600E | Mucosal | IV | ＜3 | M1b | No | No | Yes | D+T | CR | ISF | Yes | Yes | 2020/3/16 | 2020/6/1 | 2021/3/19 | 2022/11/18 | 9.70 | 12.27 | 32.57 | No | 2022/11/18 | 32.57 |
| MELA 39 | SYSUCC | 51 | Female | 0 | Normal | V600E | Non-CSD | IV | ＜3 | M1b | No | No | Yes | D+T | PR | NSF | Yes | Yes | 2021/8/11 | 2021/10/22 | 2022/1/25 | 2022/2/27 | 3.17 | 5.57 | 6.67 | Yes (within or after treatment) | 2022/1/25 | 5.57 |
| MELA 40 | SYSUCC | 49 | Female | 0 | Elevated | V600E/K | Non-CSD | IV | ≥3 | M1c | No | Yes | Yes | D+T | PR | NSF | Yes | Yes | 2018/11/21 | 2019/1/14 | 2019/5/18 | 2019/6/8 | 4.13 | 5.93 | 6.63 | Yes (within or after treatment) | 2019/5/18 | 5.93 |
| MELA 41 | SYSUCC | 58 | Female | 0 | Normal | V600E | CSD | IIIC | ＜3 | M0 | No | No | No | D+T+aPD-1 | CR | NSF | Yes | Yes | 2021/5/6 | 2021/8/9 | 2023/2/21 | 2023/6/1 | 18.70 | 21.87 | 25.20 | Yes (within or after treatment) | 2023-2-21 | 21.87 |
| MELA 42 | SYSUCC | 26 | Female | 0 | Normal | V600E/K | Non-CSD | IV | ＜3 | M1b | No | No | Yes | D+T | PR | ISF | Yes | Yes | 2017/8/24 | 2017/10/19 | 2019/6/27 | 2021/2/3 | 20.53 | 22.40 | 41.97 | No | 2021/2/3 | 41.97 |
| MELA 43 | SYSUCC | 68 | Male | 0 | Normal | V600E | Acral | IV | ＜3 | M1c | No | No | No | D+T | PR | CSF | Yes | Yes | 2021/3/31 | 2021/5/29 | 2022/8/26 | 2022/12/12 | 15.13 | 17.10 | 20.70 | No | 2022/12/12 | 20.70 |
| MELA 44 | SYSUCC | 46 | Female | 0 | Normal | V600E | Non-CSD | IV | ＜3 | M1b | No | No | Yes | D+T | SD | CSF | Yes | No | 2018/2/1 | / | 2019/2/27 | 2023/12/1 | / | 13.03 | 70.97 | Yes (within or after treatment) | 2019-2-27 | 13.03 |
| MELA 45 | SYSUCC | 47 | Female | 0 | Normal | V600E | CSD | IIIC | ＜3 | M0 | No | No | No | V+aPD-1 | SD | NSF | Yes | No | 2018/12/18 | / | 2023/2/9 | 2023/12/1 | / | 50.47 | 60.30 | No | 2023/12/1 | 60.30 |
| MELA 46 | SYSUCC | 45 | Female | 0 | Normal | V600E | Non-CSD | IIIB | ＜3 | M0 | No | No | No | V+aPD-1 | SD | NSF | Yes | No | 2019/5/22 | / | 2021/12/26 | 2023/12/1 | / | 31.63 | 55.13 | Yes (within or after treatment) | 2021-12-26 | 31.63 |
| MELA 47 | SYSUCC | 38 | Female | 0 | Normal | V600E/K | Non-CSD | IV | ＜3 | M1c | No | Yes | No | D+T+aPD-1 | CR | NSF | Yes | No | 2020/12/30 | 2021/2/4 | 2023/10/8 | 2023/12/1 | 32.53 | 33.73 | 35.53 | No | 2023/12/1 | 35.53 |
| MELA 48 | SYSUCC | 55 | Female | 1 | Normal | V600E | CSD | IV | ＜3 | M1c | No | No | No | V+aPD-1 | SD | NSF | Yes | No | 2019/11/27 |  | 2021/3/6 | 2023/3/7 | 1475.37 | 15.50 | 39.87 | Yes (within or after treatment) | 2021/3/6 | 15.50 |
| MELA 49 | SYSUCC | 56 | Female | 0 | Normal | V600E | Non-CSD | IIIB | ＜3 | M0 | No | No | No | D+T | PR | CSF | Yes | No | 2021/3/11 | 2021/5/14 | 2022/6/6 | 2023/12/1 | 12.93 | 15.07 | 33.17 | No | 2023/12/1 | 33.17 |
| MELA 50 | SYSUCC | 24 | Female | 0 | Normal | V600E/K | Non-CSD | IV | ＜3 | M1b | No | No | Yes | V+aPD-1 | PR | NSF | Yes | Yes | 2018/9/29 | 2018/11/23 | 2019/3/29 | 2019/9/12 | 4.20 | 6.03 | 11.60 | No | 2019/9/12 | 11.60 |
| MELA 51 | SYSUCC | 47 | Female | 0 | Normal | V600E/K | Non-CSD | IV | ＜3 | M1a | No | No | No | D+T+aPD-1 | SD | No progression | No | No | 2021/3/19 | / | 2023/12/1 | 2023/12/1 | / | 32.90 | 32.90 | No | 2023/12/1 | 32.90 |
| MELA 52 | SYSUCC | 36 | Male | 0 | Elevated | V600E/K | Non-CSD | IV | ＜3 | M1a | No | No | No | D+T+aPD-1 | SD | CSF | Yes | Yes | 2020/3/1 | / | 2021/9/18 | 2022/2/5 | / | 18.87 | 23.53 | Yes (within or after treatment) | 2021/9/18 | 18.87 |
| MELA 53 | SYSUCC | 29 | Female | 0 | Elevated | V600E/K | Non-CSD | IV | ≥3 | M1d | Yes | Yes | No | V+aPD-1 | PR | ISF | Yes | Yes | 2019/7/10 | 2019/10/23 | 2020/5/28 | 2021/3/24 | 7.27 | 10.77 | 20.77 | Yes (at therapy initiation) | / | / |
| MELA 54 | SYSUCC | 47 | Male | 0 | Elevated | V600E/K | CSD | IV | ≥3 | M1c | No | Yes | Yes | D+T+aPD-1 | PR | CSF | Yes | Yes | 2020/5/1 | 2021/10/13 | 2022/1/27 | 2022/10/14 | 3.53 | 21.20 | 29.87 | No | 2022/10/14 | 29.87 |
| MELA 55 | SYSUCC | 71 | Male | 0 | Normal | V600E | Non-CSD | IV | ≥3 | M1d | Yes | No | Yes | D+T | PR | ISF | Yes | Yes | 2021/12/24 | 2022/6/22 | 2022/8/18 | 2022/12/13 | 1.90 | 7.90 | 11.80 | Yes (at therapy initiation) | / | / |
| MELA 56 | SYSUCC | 70 | Female | 0 | Elevated | V600E/K | CSD | IV | ＜3 | M1a | No | No | No | V+aPD-1 | PR | CSF | Yes | Yes | 2018/9/28 | 2018/11/20 | 2019/2/25 | 2019/8/1 | 3.23 | 5.00 | 10.23 | No | 2019/8/1 | 10.23 |
| MELA 57 | SYSUCC | 59 | Male | 0 | Normal | V600E | Acral | IIIC | ＜3 | M0 | No | No | No | V+aPD-1 | CR | NSF | Yes | No | 2018/11/15 | 2019/2/14 | 2022/5/21 | 2023/12/1 | 39.73 | 42.77 | 61.40 | No | 2023/12/1 | 61.40 |
| MELA 58 | SYSUCC | 34 | Male | 0 | Normal | V600E | Mucosal | IV | ≥3 | M1c | No | Yes | No | V+aPD-1 | PR | CSF | Yes | Yes | 2018/12/21 | 2019/1/21 | 2019/9/28 | 2021/11/16 | 8.33 | 9.37 | 35.37 | Yes (within or after treatment) | 2021/8/19 | 32.40 |
| MELA 59 | SYSUCC | 80 | Male | 0 | Elevated | V600E | Non-CSD | IV | ≥3 | M1b | No | No | Yes | D+T | PR | CSF | Yes | No | 2020/9/4 | 2020/12/11 | 2021/9/14 | 2023/12/1 | 9.23 | 12.50 | 39.43 | No | 2023/12/1 | 39.43 |
| MELA 60 | SYSUCC | 65 | Female | 0 | Normal | V600E | Non-CSD | IV | ＜3 | M1b | No | No | Yes | D+T+aPD-1 | PR | No progression | No | No | 2021/5/18 | 2022/4/6 | 2023/12/1 | 2023/12/1 | 20.13 | 30.90 | 30.90 | No | 2023/12/1 | 30.90 |
| MELA 61 | SYSUCC | 49 | Female | 0 | Normal | V600E/K | Acral | IIIB/C | ＜3 | M0 | No | No | No | V+aPD-1 | SD | NSF | Yes | Yes | 2019/9/10 | / | 2021/8/18 | 2023/7/3 | / | 23.60 | 46.40 | No | 2023/7/3 | 46.40 |
| MELA 62 | SYSUCC | 60 | Male | 0 | Normal | V600E | Acral | IV | ＜3 | M1d | Yes | No | No | D+T | SD | ISF | Yes | No | 2021/11/18 | / | 2023/2/16 | 2023/12/1 | / | 15.17 | 24.77 | Yes (at therapy initiation) | / | / |
| MELA 63 | SYSUCC | 50 | Male | 0 | Elevated | V600E | Acral | IV | ＜3 | M1b | No | No | Yes | D+T | PR | CSF | Yes | No | 2020/10/20 | 2021/2/27 | 2021/9/19 | 2023/12/1 | 6.80 | 11.13 | 37.90 | No | 2023/12/1 | 37.90 |
| MELA 64 | SYSUCC | 37 | Female | 1 | Elevated | V600E | Acral | IV | ≥3 | M1d | Yes | Yes | Yes | D+T | PD | ISF | Yes | Yes | 2021/1/27 | / | 2021/4/15 | 2021/5/28 | / | 2.60 | 4.03 | Yes (at therapy initiation) | / | / |
| MELA 65 | SYSUCC | 45 | Male | 0 | Normal | V600E/K | Non-CSD | IV | ＜3 | M1d | Yes | Yes | No | V+aPD-1 | PR | No progression | No | No | 2018/12/12 | 2019/7/4 | 2023/12/1 | 2023/12/1 | 53.70 | 60.50 | 60.50 | Yes (at therapy initiation) | / | / |
| MELA 66 | SYSUCC | 52 | Male | 0 | Missing | V600E | Non-CSD | IV | ＜3 | M1c | No | Yes | No | D+T+aPD-1 | PR | NSF | Yes | No | 2020/6/23 | 2021/2/24 | 2021/8/2 | 2023/12/1 | 5.30 | 13.50 | 41.87 | Yes (within or after treatment) | 2021/8/2 | 13.50 |
| MELA 67 | SYSUCC | 38 | Male | 0 | Normal | V600E | Acral | IV | ≥3 | M1b | No | No | Yes | V+aPD-1 | PD | CSF | Yes | Yes | 2020/8/24 | / | 2021/2/25 | 2021/5/30 | / | 6.17 | 9.30 | No | 2021/5/30 | 9.30 |
| MELA 68 | SYSUCC | 48 | Female | 0 | Elevated | V600E/K | Non-CSD | IV | ＜3 | M1b | No | No | Yes | V+aPD-1 | PR | No progression | No | No | 2019/1/16 | 2019/3/1 | 2023/12/1 | 2023/12/1 | 57.87 | 59.33 | 59.33 | No | 2023/12/1 | 59.33 |
| MELA 69 | SYSUCC | 33 | Male | 0 | Normal | V600E | CSD | IV | ＜3 | M1c | No | Yes | No | D+T+aPD-1 | SD | No progression | No | No | 2021/11/24 | / | 2023/12/1 | 2023/12/1 | / | 24.57 | 24.57 | No | 2023/12/1 | 24.57 |
| MELA 70 | SYSUCC | 24 | Female | 0 | Missing | V600E | CSD | IIIC | ＜3 | M0 | No | No | No | D+T | SD | NSF | Yes | Yes | 2020/5/1 | / | 2021/4/6 | 2021/7/16 | / | 11.33 | 14.70 | No | 2021/7/16 | 14.70 |
| MELA 71 | SYSUCC | 33 | Male | 1 | Normal | V600E/K | Non-CSD | IV | ≥3 | M1d | Yes | Yes | Yes | V+aPD-1 | SD | CSF | Yes | Yes | 2018/2/11 | / | 2019/3/18 | 2019/8/28 | / | 13.33 | 18.77 | Yes (at therapy initiation) | / | / |
| MELA 72 | SYSUCC | 34 | Male | 0 | Normal | V600E/K | CSD | IV | ＜3 | M1b | No | No | Yes | V+aPD-1 | CR | No progression | No | No | 2018/12/6 | 2019/8/5 | 2023/12/1 | 2023/12/1 | 52.63 | 60.70 | 60.70 | No | 2023/12/1 | 60.70 |
| MELA 73 | SYSUCC | 54 | Female | 0 | Normal | V600E | Non-CSD | IV | ＜3 | M1c | No | Yes | No | D+T | PR | CSF | Yes | No | 2021/11/16 | 2022/3/31 | 2023/1/17 | 2023/12/1 | 9.73 | 14.23 | 24.83 | No | 2023/12/1 | 24.83 |
| MELA 74 | SYSUCC | 35 | Male | 0 | Normal | V600E/K | Non-CSD | IIIC | ＜3 | M0 | No | No | No | V | PR | NSF | Yes | No | 2017/4/10 | 2017/6/30 | 2017/9/10 | 2023/12/1 | 2.40 | 5.10 | 80.87 | Yes (within or after treatment) | 2017/9/10 | 5.10 |
| MELA 75 | SYSUCC | 49 | Female | 0 | Missing | V600E/K | Non-CSD | IV | ＜3 | M1a | No | No | No | D+T | PR | NSF | Yes | Yes | 2019/8/1 | 2020/1/9 | 2020/4/29 | 2020/6/18 | 3.70 | 9.07 | 10.73 | No | 2020/6/18 | 10.73 |
| MELA 76 | SYSUCC | 44 | Male | 0 | Normal | V600E | Mucosal | IIIB | ＜3 | M0 | No | No | No | V+aPD-1 | CR | CSF | Yes | No | 2018/12/19 | 2020/10/19 | 2023/1/16 | 2023/12/1 | 27.30 | 49.63 | 60.27 | No | 2023/12/1 | 60.27 |
| MELA 77 | SYSUCC | 67 | Female | 0 | Elevated | V600E | Non-CSD | IV | ＜3 | M1b | No | No | Yes | V | PR | NSF | Yes | Yes | 2020/5/8 | 2020/6/17 | 2020/8/13 | 2020/10/1 | 1.90 | 3.23 | 4.87 | No | 2020/10/1 | 4.87 |
| MELA 78 | SYSUCC | 63 | Female | 0 | Normal | V600E | Mucosal | IV | ＜3 | M1d | Yes | No | No | D+T+aPD-1 | SD | ISF | Yes | Yes | 2020/9/5 | / | 2021/12/8 | 2022/3/25 | / | 15.30 | 18.87 | Yes (at therapy initiation) | / | / |
| MELA 79 | SYSUCC | 50 | Female | 0 | Missing | V600E | Non-CSD | IIIB | ＜3 | M0 | No | No | No | V | PR | NSF | Yes | Yes | 2020/4/14 | 2020/9/22 | 2021/3/1 | 2022/4/8 | 5.33 | 10.70 | 24.13 | No | 2022/4/8 | 24.13 |
| MELA 80 | SYSUCC | 49 | Female | 1 | Elevated | V600E | Acral | IV | ≥3 | M1d | Yes | Yes | Yes | D+T | PD | ISF | Yes | Yes | 2021/5/17 | / | 2021/10/25 | 2022/1/7 | / | 5.37 | 7.83 | Yes (at therapy initiation) | / | / |
| MELA 81 | SYSUCC | 56 | Female | 0 | Normal | V600E/K | Mucosal | IIIC | ＜3 | M0 | No | No | No | V | PD | CSF | Yes | Yes | 2019/1/25 | / | 2019/4/1 | 2019/12/12 | / | 2.20 | 10.70 | Yes (within or after treatment) | 2019/6/1 | 4.23 |
| MELA 82 | SYSUCC | 57 | Male | 0 | Normal | V600E | Non-CSD | IV | ≥3 | M1c | No | Yes | Yes | V+aPD-1 | SD | CSF | Yes | Yes | 2019/1/10 | / | 2019/8/24 | 2019/9/23 | / | 7.53 | 8.53 | Yes (within or after treatment) | 2019/8/24 | 7.53 |
| MELA 83 | SYSUCC | 41 | Male | 1 | Normal | V600E | CSD | IV | ＜3 | M1b | No | No | Yes | V+aPD-1 | CR | NSF | Yes | No | 2018/7/2 | 2019/8/15 | 2020/10/20 | 2023/12/1 | 14.40 | 28.03 | 65.93 | No | 2023/12/1 | 65.93 |
| MELA 84 | SYSUCC | 53 | Male | 0 | Missing | V600E/K | Non-CSD | IV | ＜3 | M1d | Yes | No | No | V | PR | ISF | Yes | Yes | 2015/5/23 | 2015/6/23 | 2015/11/25 | 2016/4/15 | 5.17 | 6.20 | 10.93 | Yes (at therapy initiation) | / | / |
| MELA 85 | SYSUCC | 38 | Female | 0 | Normal | V600E/K | Non-CSD | IV | ＜3 | M1b | No | No | Yes | V+aPD-1 | PD | CSF | Yes | Yes | 2018/12/20 | / | 2019/6/1 | 2019/8/8 | / | 5.43 | 7.70 | No | 2019/8/8 | 7.70 |
| MELA 86 | SYSUCC | 68 | Male | 0 | Normal | V600E | Acral | IV | ＜3 | M1b | No | No | Yes | V+aPD-1 | PD | NSF | Yes | Yes | 2020/7/31 | / | 2021/1/6 | 2021/10/14 | / | 5.30 | 14.67 | No | 2021/10/14 | 14.67 |
| MELA 87 | SYSUCC | 29 | Female | 0 | Missing | V600E | Non-CSD | IV | ＜3 | M1d | Yes | No | Yes | D+T+aPD-1 | SD | ISF | Yes | No | 2020/8/1 | / | 2021/12/31 | 2023/12/1 | / | 17.23 | 40.57 | Yes (at therapy initiation) | / | / |
| MELA 88 | SYSUCC | 52 | Male | 0 | Normal | V600E/K | CSD | IV | ≥3 | M1c | No | No | Yes | V | PR | NSF | Yes | Yes | 2019/3/22 | 2020/1/28 | 2020/3/1 | 2020/8/5 | 1.10 | 11.50 | 16.73 | No | 2020/8/5 | 16.73 |
| MELA 89 | SYSUCC | 72 | Male | 0 | Normal | V600E/K | Non-CSD | IV | ＜3 | M1c | No | No | No | V | SD | CSF | Yes | Yes | 2019/10/18 | / | 2020/3/11 | 2020/5/14 | / | 4.83 | 6.97 | No | 2020/5/14 | 6.97 |
| MELA 90 | SYSUCC | 28 | Female | 1 | Normal | V600E | Acral | IV | ≥3 | M1d | Yes | No | Yes | D+T | PR | ISF | Yes | Yes | 2020/11/30 | 2021/3/13 | 2021/4/28 | 2021/7/29 | 1.53 | 4.97 | 8.03 | Yes (at therapy initiation) | / | / |
| MELA 91 | SYSUCC | 29 | Male | 0 | Normal | V600E | CSD | IIIB/C | ＜3 | M0 | No | No | No | D+T | SD | CSF | Yes | No | 2022/1/25 | / | 2022/7/5 | 2023/12/1 | / | 5.37 | 22.50 | No | 2023/12/1 | 22.50 |
| MELA 92 | SYSUCC | 29 | Male | 0 | Elevated | V600E | Non-CSD | IV | ＜3 | M1d | Yes | No | No | D+T+aPD-1 | CR | No progression | No | No | 2020/4/2 | 2020/6/12 | 2023/12/1 | 2023/12/1 | 42.23 | 44.60 | 44.60 | Yes (at therapy initiation) | / | / |
| MELA 93 | SYSUCC | 75 | Male | 0 | Elevated | V600E | Non-CSD | IV | ≥3 | M1c | No | Yes | Yes | V | SD | Death without failure | Yes | Yes | 2020/1/11 | / | 2020/10/18 | 2020/10/18 | / | 9.37 | 9.37 | No | 2020/10/18 | 9.37 |
| MELA 94 | SYSUCC | 36 | Female | 0 | Normal | V600E/K | Non-CSD | IIIC | ＜3 | M0 | No | No | No | D+T+aPD-1 | CR | No progression | No | No | 2021/6/5 | 2021/9/8 | 2023/12/1 | 2023/12/1 | 27.13 | 30.30 | 30.30 | No | 2023/12/1 | 30.30 |
| MELA 95 | SYSUCC | 39 | Female | 1 | Elevated | V600E/K | CSD | IV | ≥3 | M1d | Yes | Yes | Yes | D+T | SD | ISF | Yes | Yes | 2020/3/18 | / | 2020/8/6 | 2021/5/25 | / | 4.70 | 14.43 | Yes (at therapy initiation) | / | / |
| MELA 96 | SYSUCC | 21 | Female | 0 | Normal | V600E | Non-CSD | IV | ＜3 | M1a | No | No | No | D+T+aPD-1 | PR | No progression | No | No | 2021/3/5 | 2021/4/28 | 2023/12/1 | 2023/12/1 | 31.57 | 33.37 | 33.37 | No | 2023/12/1 | 33.37 |
| MELA 97 | SYSUCC | 47 | Female | 0 | Normal | V600E | Non-CSD | IV | ≥3 | M1c | No | No | Yes | D+T+aPD-1 | PR | CSF | Yes | Yes | 2020/10/27 | 2021/2/26 | 2022/10/17 | 2023/3/21 | 19.93 | 24.00 | 29.17 | Yes (within or after treatment) | 2022/10/28 | 24.37 |
| MELA 98 | SYSUCC | 66 | Male | 0 | Normal | V600E | Non-CSD | IV | ＜3 | M1c | No | Yes | Yes | D+T+aPD-1 | PR | No progression | No | No | 2021/4/6 | 2021/6/24 | 2023/12/1 | 2023/12/1 | 29.67 | 32.30 | 32.30 | No | 2023/12/1 | 32.30 |
| MELA 99 | SYSUCC | 47 | Male | 0 | Normal | V600E | Non-CSD | IV | ＜3 | M1c | No | No | No | D+T | PR | CSF | Yes | Yes | 2021/5/26 | 2021/9/22 | 2021/12/26 | 2022/6/13 | 3.17 | 7.13 | 12.77 | Yes (within or after treatment) | 2021/12/26 | 7.13 |
| MELA 100 | SYSUCC | 37 | Male | 0 | Missing | V600E | Non-CSD | IV | ≥3 | M1c | No | Yes | Yes | D+T | PR | ISF | Yes | Yes | 2021/9/1 | 2021/11/18 | 2022/4/20 | 2022/7/10 | 5.10 | 7.70 | 10.40 | No | 2022/7/10 | 10.40 |
| MELA 101 | SYSUCC | 40 | Female | 0 | Normal | V600E/K | Non-CSD | IV | ≥3 | M1c | No | Yes | Yes | V | SD | CSF | Yes | Yes | 2020/1/9 | / | 2020/8/1 | 2021/8/8 | / | 6.83 | 19.23 | Yes (within or after treatment) | 2020-9-14 | 8.30 |
| MELA 102 | SYSUCC | 60 | Female | 0 | Normal | V600E/K | CSD | IV | ＜3 | M1a | No | No | No | V+aPD-1 | CR | NSF | Yes | No | 2018/12/3 | 2020/12/1 | 2022/5/24 | 2023/12/1 | 17.97 | 42.27 | 60.80 | No | 2023/12/1 | 60.80 |
| MELA 103 | SYSUCC | 65 | Male | 1 | Missing | V600E | Acral | IV | ≥3 | M1d | Yes | No | Yes | V+aPD-1 | SD | Death without failure | Yes | Yes | 2020/5/1 | / | 2021/4/15 | 2021/4/15 | / | 11.63 | 11.63 | Yes (at therapy initiation) | / | / |
| MELA 104 | SYSUCC | 67 | Female | 1 | Normal | V600E/K | Non-CSD | IV | ＜3 | M1d | Yes | No | No | V+aPD-1 | PR | Death without failure | Yes | Yes | 2017/10/29 | 2018/3/19 | 2018/6/11 | 2018/6/11 | 2.80 | 7.50 | 7.50 | Yes (at therapy initiation) | / | / |
| MELA 105 | SYSUCC | 51 | Male | 0 | Elevated | V600E/K | Non-CSD | IV | ≥3 | M1d | Yes | No | Yes | V | PR | CSF | Yes | Yes | 2018/5/28 | 2018/7/10 | 2018/11/29 | 2018/12/20 | 4.73 | 6.17 | 6.87 | Yes (at therapy initiation) | / | / |
| MELA 106 | SYSUCC | 32 | Male | 0 | Normal | V600E/K | CSD | IV | ＜3 | M1b | No | No | Yes | D+T+aPD-1 | PD | CSF | Yes | Yes | 2017/8/16 | / | 2018/12/30 | 2020/10/13 | / | 16.70 | 38.47 | No | 2020/10/13 | 38.47 |
| MELA 107 | SYSUCC | 46 | Female | 0 | Elevated | V600E | Non-CSD | IIIC | ＜3 | M0 | No | No | No | D+T+aPD-1 | SD | ISF | Yes | Yes | 2018/9/1 | / | 2021/5/15 | 2021/5/15 | / | 32.90 | 32.90 | No | 2021/5/15 | 32.90 |
| MELA 108 | SYSUCC | 55 | Male | 0 | Normal | V600E | CSD | IV | ＜3 | M1d | Yes | No | No | D+T+aPD-1 | PR | NSF | Yes | No | 2021/8/11 | 2021/12/2 | 2022/10/12 | 2023/12/1 | 10.47 | 14.23 | 28.07 | Yes (at therapy initiation) | / | / |
| MELA 109 | SYSUCC | 56 | Male | 1 | Elevated | V600E/K | Mucosal | IV | ≥3 | M1c | No | Yes | No | V | SD | Death without failure | Yes | Yes | 2019/1/25 | / | 2019/6/1 | 2019/6/1 | / | 4.23 | 4.23 | No | 2019/6/1 | 4.23 |
| MELA 110 | SYSUCC | 34 | Male | 0 | Normal | V600E/K | CSD | IV | ＜3 | M1c | No | No | Yes | V | PR | CSF | Yes | Yes | 2020/1/2 | 2020/4/22 | 2020/7/27 | 2020/12/8 | 3.20 | 6.90 | 11.37 | Yes (within or after treatment) | 2020/7/27 | 6.90 |
| MELA 111 | SYSUCC | 30 | Male | 0 | Normal | V600E/K | Non-CSD | IIIB | ＜3 | M0 | No | No | No | D+T | PR | No progression | No | No | 2020/4/29 | 2020/7/31 | 2023/12/1 | 2023/12/1 | 40.60 | 43.70 | 43.70 | No | 2023/12/1 | 43.70 |
| MELA 112 | SYSUCC | 66 | Female | 0 | Normal | V600E | Acral | IIID | ＜3 | M0 | No | No | No | D+T | SD | CSF | Yes | Yes | 2021/8/1 | / | 2022/11/7 | 2022/12/11 | / | 15.43 | 16.57 | No | 2022/12/11 | 16.57 |
| MELA 113 | SYSUCC | 48 | Male | 1 | Elevated | V600E | Non-CSD | IV | ≥3 | M1d | Yes | Yes | Yes | V | PD | CSF | Yes | Yes | 2020/12/30 | / | 2021/3/11 | 2021/6/5 | / | 2.37 | 5.23 | Yes (at therapy initiation) | / | / |
| MELA 114 | SYSUCC | 37 | Male | 0 | Elevated | V600E | Non-CSD | IV | ≥3 | M1c | No | Yes | No | D+T | SD | ISF | Yes | No | 2021/6/1 | / | 2021/12/13 | 2022/5/10 | / | 6.50 | 11.43 | No | 2022-5-10 | 11.43 |
| MELA 115 | SYSUCC | 63 | Female | 0 | Elevated | V600E | Non-CSD | IV | ≥3 | M1c | No | No | No | D+T | SD | No progression | No | No | 2022/4/18 | / | 2023/12/1 | 2023/12/1 | / | 19.73 | 19.73 | No | 2023/12/1 | 19.73 |
| MELA 116 | SYSUCC | 59 | Male | 1 | Normal | V600E | Non-CSD | IV | ＜3 | M1d | Yes | No | No | V | PD | NSF | Yes | Yes | 2018/8/30 | / | 2018/12/24 | 2019/6/13 | / | 3.87 | 9.57 | Yes (at therapy initiation) | / | / |
| MELA 117 | SYSUCC | 56 | Male | 0 | Elevated | V600E/K | Acral | IV | ≥3 | M1c | No | Yes | Yes | D+T+aPD-1 | PR | NSF | Yes | No | 2020/10/16 | 2021/4/20 | 2023/5/7 | 2023/12/1 | 24.90 | 31.10 | 38.03 | No | 2023/12/1 | 38.03 |
| MELA 118 | SYSUCC | 28 | Female | 0 | Normal | V600E/K | Mucosal | IV | ＜3 | M1a | No | No | No | V | SD | NSF | Yes | Yes | 2020/1/14 | / | 2020/10/31 | 2020/12/9 | / | 9.70 | 11.00 | No | 2020/12/9 | 11.00 |
| MELA 119 | SYSUCC | 50 | Male | 1 | Normal | V600E/K | Non-CSD | IV | ≥3 | M1b | No | No | Yes | V+aPD-1 | SD | NSF | Yes | Yes | 2019/9/29 | / | 2020/4/9 | 2021/7/20 | / | 6.43 | 22.00 | Yes (within or after treatment) | 2020-4-9 | 6.43 |
| MELA 120 | SYSUCC | 37 | Female | 0 | Normal | V600E | Acral | IV | ≥3 | M1d | Yes | No | Yes | D+T | SD | Death without failure | Yes | Yes | 2021/12/30 | / | 2022/6/1 | 2022/6/1 | / | 5.10 | 5.10 | Yes (at therapy initiation) | / | / |
| MELA 121 | SYSUCC | 56 | Male | 1 | Normal | V600E/K | Acral | IV | ＜3 | M1d | Yes | No | No | V | SD | NSF | Yes | Yes | 2019/10/29 | / | 2020/2/28 | 2020/6/11 | / | 4.07 | 7.53 | Yes (at therapy initiation) | / | / |
| MELA 122 | SYSUCC | 36 | Female | 0 | Normal | V600E | Non-CSD | IV | ＜3 | M1a | No | No | No | D+T+aPD-1 | PR | CSF | Yes | Yes | 2020/7/7 | 2020/10/20 | 2021/11/6 | 2022/4/13 | 12.73 | 16.23 | 21.50 | Yes (within or after treatment) | 2021/11/6 | 16.23 |
| MELA 123 | SYSUCC | 57 | Male | 0 | Normal | V600E | Non-CSD | IV | ≥3 | M1b | No | No | Yes | V+aPD-1 | PR | NSF | Yes | Yes | 2019/7/17 | 2019/10/25 | 2020/8/6 | 2021/12/17 | 9.53 | 12.87 | 29.47 | No | 2021/12/17 | 29.47 |
| MELA 124 | SYSUCC | 78 | Female | 1 | Normal | V600E | CSD | IV | ＜3 | M1d | Yes | No | No | D+T+aPD-1 | SD | CSF | Yes | No | 2018/4/5 | / | 2019/4/3 | 2023/12/1 | / | 12.10 | 68.87 | Yes (at therapy initiation) | / | / |
| MELA 125 | SYSUCC | 57 | Male | 0 | Elevated | V600E/K | Non-CSD | IV | ≥3 | M1c | No | No | Yes | V | PR | NSF | Yes | Yes | 2019/6/12 | 2020/1/3 | 2020/6/24 | 2020/11/11 | 5.77 | 12.60 | 17.27 | Yes (within or after treatment) | 2020/6/27 | 12.70 |
| MELA 126 | SYSUCC | 36 | Male | 0 | Normal | V600E | Acral | IV | ＜3 | M1c | No | Yes | Yes | D+T | PR | CSF | Yes | No | 2022/1/17 | 2022/3/25 | 2022-11-17 | 2022/12/16 | 7.90 | 10.13 | 11.10 | Yes (within or after treatment) | 2022-10-14 | 9.00 |
| MELA 127 | SYSUCC | 60 | Female | 0 | Normal | V600E | Non-CSD | IV | ＜3 | M1b | No | No | Yes | D+T | PR | CSF | Yes | No | 2020/11/28 | 2021/3/7 | 2021/7/8 | 2023/12/1 | 4.10 | 7.40 | 36.60 | Yes (within or after treatment) | 2021-7-8 | 7.40 |
| MELA 128 | SYSUCC | 61 | Male | 0 | Normal | V600E/K | CSD | IV | ≥3 | M1b | No | No | Yes | D+T | PR | CSF | Yes | Yes | 2021/4/20 | 2021/7/25 | 2022/10/12 | 2023/2/2 | 14.80 | 18.00 | 21.77 | Yes (within or after treatment) | 2022-10-12 | 18.00 |
| MELA 129 | SYSUCC | 49 | Male | 0 | Normal | V600E | Non-CSD | IIIB/C | ＜3 | M0 | No | No | No | V+aPD-1 | SD | NSF | Yes | Yes | 2019/4/23 | / | 2020/11/6 | 2020/12/25 | / | 18.77 | 20.40 | No | 2020/12/25 | 20.40 |
| MELA 130 | SYSUCC | 27 | Female | 0 | Normal | V600E/K | Non-CSD | IV | ＜3 | M1b | No | No | Yes | V | SD | CSF | Yes | Yes | 2017/11/30 | / | 2018/8/23 | 2018/10/2 | / | 8.87 | 10.20 | Yes (within or after treatment) | 2018/8/23 | 8.87 |
| MELA 131 | SYSUCC | 57 | Female | 0 | Normal | V600E/K | Acral | IV | ≥3 | M1c | No | Yes | Yes | D+T+aPD-1 | PR | CSF | Yes | No | 2020/8/29 | 2020/10/19 | 2022/3/30 | 2023/12/1 | 17.57 | 19.27 | 39.63 | No | 2023/12/1 | 39.63 |
| MELA 132 | SYSUCC | 55 | Male | 0 | Normal | V600E/K | Non-CSD | IV | ≥3 | M1d | Yes | No | Yes | D+T+aPD-1 | SD | ISF | Yes | No | 2019/12/5 | / | 2021/12/27 | 2023/12/1 | / | 25.10 | 48.57 | Yes (at therapy initiation) | / | / |
| MELA 133 | SYSUCC | 33 | Female | 0 | Elevated | V600E | Non-CSD | IV | ＜3 | M1a | No | No | No | D+T+aPD-1 | PD | CSF | Yes | No | 2021/8/9 | / | 2022/2/9 | 2023/12/1 | / | 6.13 | 28.13 | No | 2023/12/1 | 28.13 |
| MELA 134 | SYSUCC | 53 | Female | 0 | Normal | V600E | Non-CSD | IIIC | ＜3 | M0 | No | No | No | D+T | PR | NSF | Yes | No | 2021/4/30 | 2022/2/22 | 2022/7/26 | 2023/12/1 | 5.13 | 15.07 | 31.50 | No | 2023/12/1 | 31.50 |
| MELA 135 | SYSUCC | 49 | Male | 0 | Normal | V600E | Non-CSD | IV | ≥3 | M1d | Yes | No | Yes | D+T | PR | ISF | Yes | Yes | 2021/4/16 | 2021/6/23 | 2021/12/8 | 2023/2/22 | 5.60 | 7.87 | 22.57 | Yes (at therapy initiation) | / | / |
| MELA 136 | SYSUCC | 70 | Female | 0 | Normal | V600E | CSD | IV | ＜3 | M1d | Yes | No | No | D+T | SD | ISF | Yes | No | 2022/3/24 | / | 2022/10/13 | 2023/12/1 | / | 6.77 | 20.57 | Yes (at therapy initiation) | / | / |
| MELA 137 | SYSUCC | 53 | Male | 0 | Normal | V600E/K | Non-CSD | IV | ＜3 | M1a | No | No | No | V+aPD-1 | CR | NSF | Yes | No | 2018/6/1 | 2018/12/10 | 2022/3/29 | 2023/12/1 | 40.17 | 46.57 | 66.97 | No | 2023/12/1 | 66.97 |
| MELA 138 | SYSUCC | 52 | Male | 0 | Normal | V600E/K | Non-CSD | IV | ＜3 | M1a | No | No | No | V | SD | CSF | Yes | Yes | 2018/9/2 | / | 2019/4/3 | 2019/5/24 | / | 7.10 | 8.80 | No | 2019/5/24 | 8.80 |
| MELA 139 | SYSUCC | 40 | Male | 0 | Elevated | V600E/K | Non-CSD | IV | ≥3 | M1d | Yes | Yes | Yes | V | SD | CSF | Yes | Yes | 2017/8/29 | / | 2018/2/5 | 2018/2/26 | / | 5.33 | 6.03 | Yes (at therapy initiation) | / | / |
| MELA 140 | SYSUCC | 49 | Female | 0 | Normal | V600E | Non-CSD | IIIB/C | ＜3 | M0 | No | No | No | D+T+aPD-1 | PR | CSF | Yes | No | 2022/1/7 | 2022/8/19 | 2023/2/4 | 2023/12/1 | 5.63 | 13.10 | 23.10 | No | 2023/12/1 | 23.10 |
| MELA 141 | SYSUCC | 29 | Female | 0 | Normal | V600E | Non-CSD | IV | ≥3 | M1c | No | Yes | No | D+T+aPD-1 | PR | NSF | Yes | Yes | 2021/8/27 | 2022/1/27 | 2022/5/5 | 2023/1/6 | 3.27 | 8.37 | 16.57 | Yes (within or after treatment) | 2022-5-5 | 8.37 |
| MELA 142 | SYSUCC | 47 | Female | 1 | Normal | V600E/K | Acral | IV | ＜3 | M1c | No | No | No | V+aPD-1 | SD | ISF | Yes | Yes | 2019/7/25 | / | 2020/10/16 | 2021/4/10 | / | 14.97 | 20.83 | No | 2021/4/10 | 20.83 |
| MELA 143 | SYSUCC | 52 | Male | 0 | Elevated | V600E/K | Non-CSD | IV | ＜3 | M1b | No | No | Yes | V | PR | Death without failure | Yes | Yes | 2019/10/11 | 2019/12/4 | 2020/3/27 | 2020/3/27 | 3.80 | 5.60 | 5.60 | No | 2020/3/27 | 5.60 |
| MELA 144 | SYSUCC | 24 | Female | 0 | Normal | V600E | Non-CSD | IIID | ＜3 | M0 | No | No | No | D+T+aPD-1 | PR | No progression | No | No | 2020/5/28 | 2020/7/27 | 2023/12/1 | 2023/12/1 | 40.73 | 42.73 | 42.73 | No | 2023/12/1 | 42.73 |
| MELA 145 | SYSUCC | 30 | Female | 0 | Normal | V600E | Non-CSD | IV | ＜3 | M1a | No | No | No | D+T | CR | ISF | Yes | No | 2021/12/22 | 2022/4/6 | 2023/2/13 | 2023/12/1 | 10.43 | 13.93 | 23.63 | No | 2023/12/1 | 23.63 |
| MELA 146 | SYSUCC | 43 | Female | 0 | Normal | V600E | Acral | IV | ＜3 | M1a | No | No | No | V+aPD-1 | PR | No progression | No | No | 2020/6/10 | 2020/8/10 | 2023/12/1 | 2023/12/1 | 40.27 | 42.30 | 42.30 | No | 2023/12/1 | 42.30 |
| MELA 147 | SYSUCC | 44 | Female | 0 | Normal | V600E | Non-CSD | IV | ＜3 | M1b | No | No | Yes | V | CR | NSF | Yes | No | 2019/8/5 | 2020/3/31 | 2021/1/10 | 2022/11/14 | 9.50 | 17.47 | 39.90 | No | 2022/11/14 | 39.90 |
| MELA 148 | SYSUCC | 64 | Male | 0 | Normal | V600E/K | Non-CSD | IV | ≥3 | M1d | Yes | Yes | Yes | D+T+aPD-1 | PR | ISF | Yes | Yes | 2019/5/14 | 2020/3/18 | 2021/5/20 | 2021/7/10 | 14.27 | 24.57 | 26.27 | Yes (at therapy initiation) | / | / |
| MELA 149 | SYSUCC | 32 | Female | 0 | Missing | V600E | CSD | IV | ≥3 | M1c | No | No | Yes | V | PR | CSF | Yes | Yes | 2018/2/11 | 2018/5/26 | 2018/12/8 | 2020/4/6 | 6.53 | 10.00 | 26.17 | No | 2020/4/6 | 26.17 |
| MELA 150 | SYSUCC | 40 | Male | 0 | Elevated | V600E | Non-CSD | IIIC/D | ＜3 | M0 | No | No | No | V | PR | Death without failure | Yes | Yes | 2018/7/17 | 2018/8/1 | 2019/1/15 | 2019/1/15 | 5.57 | 6.07 | 6.07 | No | 2019/1/15 | 6.07 |
| MELA 151 | SYSUCC | 59 | Female | 0 | Normal | V600E | CSD | IV | ＜3 | M1c | No | No | No | V | PR | ISF | Yes | Yes | 2017/9/7 | 2017/12/8 | 2018/3/16 | 2019/2/27 | 3.27 | 6.33 | 17.93 | No | 2019/2/27 | 17.93 |
| MELA 152 | SYSUCC | 39 | Female | 0 | Normal | V600E | CSD | IV | ＜3 | M1b | No | No | Yes | V+aPD-1 | CR | NSF | Yes | Yes | 2018/7/15 | 2018/11/16 | 2019/7/25 | 2022/2/17 | 8.37 | 12.50 | 43.77 | No | 2022/2/17 | 43.77 |
| MELA 153 | SYSUCC | 54 | Female | 0 | Normal | V600E/K | Acral | IV | ＜3 | M1b | No | No | Yes | V | SD | ISF | Yes | Yes | 2019/12/1 | / | 2020/4/3 | 2020/9/15 | / | 4.13 | 9.63 | No | 2020/9/15 | 9.63 |
| MELA 154 | SHENZHEN | 35 | Female | 0 | Normal | V600E | Non-CSD | IV | ＜3 | M1b | No | No | Yes | D+T+aPD-1 | CR | No progression | No | No | 2022/11/1 | 2022/12/1 | 2023/12/1 | 2023/12/1 | 12.17 | 13.17 | 13.17 | No | 2023/12/1 | 13.17 |
| MELA 155 | SHENZHEN | 65 | Female | 0 | Elevated | V600E | Non-CSD | IV | ≥3 | M1d | Yes | Yes | Yes | V | PD | ISF | Yes | No | 2023/4/24 | / | 2023/11/1 | 2023/12/1 | / | 6.37 | 7.37 | Yes (at therapy initiation) | / | / |
| MELA 156 | SHENZHEN | 57 | Female | 0 | Normal | V600E | Non-CSD | IV | ≥3 | M1c | No | No | Yes | D+T+aPD-1 | PR | ISF | Yes | Yes | 2021/3/18 | 2021/4/27 | 2021/7/14 | 2021/9/8 | 2.60 | 3.93 | 5.80 | No | 2021/9/8 | 5.80 |
| MELA 157 | SHENZHEN | 40 | Female | 1 | Normal | V600E | Mucosal | IV | ≥3 | M1d | Yes | Yes | Yes | D+T | PR | CSF | Yes | No | 2022/1/16 | 2022/2/24 | 2023/10/17 | 2023/12/1 | 20.00 | 21.30 | 22.80 | Yes (at therapy initiation) | / | / |
| MELA 158 | SHENZHEN | 51 | Female | 0 | Missing | V600E | Mucosal | IV | ＜3 | M1b | No | No | Yes | D+T | SD | ISF | Yes | No | 2021/7/15 | / | 2023/4/25 | 2023/12/1 | / | 21.63 | 28.97 | No | 2023/12/1 | 28.97 |
| MELA 159 | SHENZHEN | 61 | Female | 0 | Elevated | V600E | Non-CSD | IV | ＜3 | M1d | Yes | No | No | D+T+aPD-1 | PD | CSF | Yes | No | 2023/2/17 | / | 2023/11/9 | 2023/12/1 | / | 8.83 | 9.57 | Yes (at therapy initiation) | / | / |
| MELA 160 | SHENZHEN | 57 | Male | 0 | Missing | V600E | Non-CSD | IV | ＜3 | M1b | No | No | Yes | D+T | SD | NSF | Yes | No | 2022/11/13 | / | 2023/11/29 | 2023/12/1 | / | 12.70 | 12.77 | No | 2023/12/1 | 12.77 |
| MELA 161 | SHENZHEN | 32 | Female | 0 | Normal | V600E | Non-CSD | IV | ≥3 | M1c | No | No | Yes | D+T+aPD-1 | PR | ISF | Yes | No | 2022/12/6 | 2023/2/15 | 2023/6/13 | 2023/12/1 | 3.93 | 6.30 | 12.00 | No | 2023/12/1 | 12.00 |
| MELA 162 | SHENZHEN | 41 | Female | 0 | Normal | V600E | Non-CSD | IV | ≥3 | M1c | No | Yes | Yes | D+T+aPD-1 | PR | ISF | Yes | No | 2021/1/30 | 2021/5/15 | 2023/7/13 | 2023/12/1 | 26.30 | 29.80 | 34.50 | No | 2023/12/1 | 34.50 |
| MELA 163 | SHENZHEN | 32 | Female | 1 | Normal | V600E | Non-CSD | IV | ≥3 | M1c | No | Yes | Yes | D+T | PR | NSF | Yes | Yes | 2020/8/27 | 2020/10/15 | 2021/6/25 | 2021/8/20 | 8.43 | 10.07 | 11.93 | No | 2021/8/20 | 11.93 |
| MELA 164 | SHENZHEN | 39 | Female | 0 | Missing | V600E | Non-CSD | IV | ＜3 | M1a | No | No | No | D+T+aPD-1 | SD | CSF | Yes | Yes | 2021/11/26 | / | 2022/11/11 | 2022/11/25 | / | 11.67 | 12.13 | No | 2022/11/25 | 12.13 |
| MELA 165 | SHENZHEN | 49 | Female | 0 | Missing | V600E | Mucosal | IV | ≥3 | M1c | No | Yes | No | D+T+aPD-1 | SD | NSF | Yes | Yes | 2021/9/17 | / | 2023/8/7 | 2023/8/21 | / | 22.97 | 23.43 | Yes (within or after treatment) | 2022/8/3 | 10.67 |
| MELA 166 | SHENZHEN | 38 | Female | 0 | Normal | V600E | Non-CSD | IV | ＜3 | M1b | No | No | Yes | D+T+aPD-1 | SD | Death without failure | Yes | No | 2022/5/15 | / | 2023/11/28 | 2023/12/1 | / | 18.73 | 18.83 | No | 2023/12/1 | 18.83 |
| MELA 167 | YUNNAN | 41 | Female | 0 | Normal | V600E | Non-CSD | IV | ≥3 | M1d | No | No | Yes | V | SD | NSF | Yes | Yes | 2020/4/22 | 2020/5/18 | 2021/2/28 | 2021/4/1 | 9.53 | 10.40 | 11.47 | Yes (within or after treatment) | 2021/2/28 | 10.40 |
| MELA 168 | YUNNAN | 67 | Female | 0 | Elevated | V600E | Mucosal | IV | ＜3 | M1a | No | No | No | D+T | SD | CSF | Yes | No | 2021/7/6 | / | 2022/3/23 | 2023/12/1 | / | 8.67 | 29.27 | Yes (within or after treatment) | 2022/3/23 | 8.67 |
| MELA 169 | YUNNAN | 58 | Female | 0 | Normal | V600E | CSD | IIIC | ＜3 | M0 | No | No | No | D+T | PR | No progression | No | No | 2022/9/15 | 2023/3/15 | 2023/12/1 | 2023/12/1 | 8.70 | 14.73 | 14.73 | No | 2023/12/1 | 14.73 |
| MELA 170 | YUNNAN | 49 | Female | 0 | Normal | V600E | CSD | IIIC | ≥3 | M0 | No | No | No | D+T | SD | No progression | No | No | 2021/7/6 | / | 2023/12/1 | 2023/12/1 | / | 29.27 | 29.27 | No | 2023/12/1 | 29.27 |
| MELA 171 | YUNNAN | 66 | Male | 0 | Normal | V600E | Acral | IIIC | ≥3 | M0 | No | No | No | V | PR | NSF | Yes | Yes | 2020/3/30 | 2020/6/8 | 2021/2/1 | 2021/4/1 | 7.93 | 10.27 | 12.23 | No | 2021/4/1 | 12.23 |
| MELA 172 | YUNNAN | 68 | Male | 1 | Elevated | V600E | Non-CSD | IV | ≥3 | M1d | No | Yes | Yes | V | PD | Death without failure | Yes | Yes | 2020/7/24 | / | 2020/8/25 | 2020/8/25 | / | 1.07 | 1.07 | No | 2020/8/25 | 1.07 |
| MELA 173 | YUNNAN | 57 | Male | 0 | Elevated | V600E | CSD | IIIC | ≥3 | M0 | No | No | No | D+T | PD | CSF | Yes | Yes | 2021/12/11 | / | 2022/4/8 | 2022/5/1 | / | 3.93 | 4.70 | Yes (within or after treatment) | 2022/4/8 | 3.93 |
| MELA 174 | YUNNAN | 44 | Female | 0 | Elevated | V600E | Non-CSD | IV | ＜3 | M1c | Yes | No | Yes | D+T | SD | ISF | Yes | No | 2022/10/7 | / | 2023/4/9 | 2023/12/1 | / | 6.13 | 14.00 | Yes (at therapy initiation) | / | / |
| MELA 175 | YUNNAN | 57 | Female | 0 | Elevated | V600E | Mucosal | IV | ≥3 | M1b | No | Yes | No | V | PR | Death without failure | Yes | Yes | 2020/2/29 | 2020/4/15 | 2020/7/24 | 2020/7/24 | 3.33 | 4.87 | 4.87 | No | 2020/7/24 | 4.87 |
| MELA 176 | YUNNAN | 50 | Female | 0 | Normal | V600E | Acral | IIIC | ≥3 | M0 | No | No | No | D+T | PR | No progression | No | No | 2022/8/24 | 2022/10/31 | 2023/12/1 | 2023/12/1 | 13.20 | 15.47 | 15.47 | No | 2023/12/1 | 15.47 |
| MELA 177 | YUNNAN | 36 | Female | 0 | Normal | V600E | Non-CSD | IV | ≥3 | M1a | No | No | No | V | PR | ISF | Yes | No | 2020/9/14 | 2021/3/31 | 2021/9/14 | 2023/12/1 | 5.57 | 12.17 | 39.10 | No | 2023/12/1 | 39.10 |
| MELA 178 | YUNNAN | 49 | Male | 0 | Normal | V600E | CSD | IIID | ≥3 | M0 | No | No | No | D+T | PD | NSF | Yes | No | 2023/4/10 | / | 2023/8/14 | 2023/12/1 | / | 4.20 | 7.83 | No | 2023/12/1 | 7.83 |

V, vemurafenib; D, dabrafenib; T, trametinib; aPD-1, anti-PD-1 antibody; ECOG PS, Eastern Cooperative Oncology Group Performance Status; LDH, lactate dehydrogenases; CSD, chronic sun-induced damage; ISF, initial-site failure; NSF, new-site failure; CSF, combined-site failure; CR, complete response; PR, partial response; SD, stable disease; PD, progressive disease.

**Table S2. Follow-up time of patients stratified by first-line therapy.**

| **Treatment options** | **Follow-up time, months, median (IQR)** |
| --- | --- |
| D+T (n=51) | 24.8 (20.6–37.9) |
| D+T+aPD-1 (n=43) | 32.9 (28.1–40.6) |
| V (n=45) | 39.9 (39.1–80.9) |
| V+aPD-1 (n=39) | 60.3 (43.4–61.4) |
| Total (n=178) | 37.9 (26.1–55.1) |

D, dabrafenib; T, trametinib; V, vemurafenib; aPD-1, anti-PD-1 antibody.

**Table S3. Treatment response of patients stratified by first-line therapy.**

| **Overall response, n (%)** | **MAPKi** | |  | **MAPKi + aPD-1** | | **P value** |
| --- | --- | --- | --- | --- | --- | --- |
|  | **V**  **(n=45)** | **D+T**  **(n=51)** |  | **V+aPD-1 (n=39)** | **D+T+aPD-1 (n=43)** |  |
| Objective response rate | 21 (46.7) | 28 (54.9) |  | 20 (51.3) | 28 (65.1) | 0.357 |
| Disease control rate | 39 (86.6) | 46 (90.2) |  | 36 (92.3) | 40 (93.0) | 0.787 |
| Overall CR | 1 (2.2) | 3 (5.9) |  | 7 (17.9) | 5 (11.6) | 0.064 |
| Overall PR | 20 (44.4) | 25 (49.0) |  | 13 (33.3) | 23 (53.5) | 0.295 |
| Overall SD | 18 (40.0) | 18 (35.3) |  | 16 (41.0) | 12 (27.9) | 0.578 |
| Overall PD | 6 (13.3) | 5 (9.8) |  | 3 (7.7) | 3 (7.0) | 0.787 |

V, vemurafenib; D, dabrafenib; T, trametinib; aPD-1, anti-PD-1 antibody; CR, complete response; PR, partial response; SD, stable disease; PD, progressive disease.

**Table S4. Intergroup comparison of treatment response.**

| **Overall response, n (%)** | **V**  **(n=45)** | **V+aPD-1 (n=39)** | **P value**  **(V/ V+aPD-1)** | **D+T**  **(n=51)** | **D+T+aPD-1 (n=43)** | **P value**  **(D+T/**  **D+T+aPD-1)** |
| --- | --- | --- | --- | --- | --- | --- |
| Objective response rate | 21 (46.7) | 20 (51.3) | 0.673 | 28 (54.9) | 28 (65.1) | 0.315 |
| Disease control rate | 39 (86.6) | 36 (92.3) | 0.631 | 46 (90.2) | 40 (93.0) | 0.906 |
| Overall CR | 1 (2.2) | 7 (17.9) | **0.038** | 3 (5.9) | 5 (11.6) | 0.533 |
| Overall PR | 20 (44.4) | 13 (33.3) | 0.298 | 25 (49.0) | 23 (53.5) | 0.666 |
| Overall SD | 18 (40.0) | 16 (41.0) | 0.924 | 18 (35.3) | 12 (27.9) | 0.444 |
| Overall PD | 6 (13.3) | 3 (7.7) | 0.631 | 5 (9.8) | 3 (7.0) | 0.906 |

P values <0.05 in bold are statistically significant.

V, vemurafenib; D, dabrafenib; T, trametinib; aPD-1, anti-PD-1 antibody; CR, complete response; PR, partial response; SD, stable disease; PD, progressive disease.

**Table S5. Univariable analysis of prognostic factors correlated with PFS.**

| **Characteristics** | **Total** | **Events** | **mPFS (months)** | **HR (95%CI)** | **P value** |
| --- | --- | --- | --- | --- | --- |
|  |  |  |  |  |  |
| **Age** |  |  |  |  |  |
| ＜60 | 141 | 121 | 11.7 | Reference |  |
| ≥60 | 37 | 32 | 10.3 | 1.199 (0.810 to 1.774) | 0.365 |
| **Gender** |  |  |  |  |  |
| Male | 76 | 65 | 11.1 | Reference |  |
| Female | 102 | 88 | 11.7 | 1.040 (0.753 to 1.435) | 0.814 |
| **ECOG PS** |  |  |  |  |  |
| 0 | 150 | 127 | 12.3 | Reference |  |
| 1-2 | 28 | 26 | 5.4 | 2.017 (1.317 to 3.090) | **0.001** |
| **Serum LDH** |  |  |  |  |  |
| Normal | 120 | 100 | 13.2 | Reference |  |
| Elevated | 41 | 37 | 6.1 | 1.876 (1.284 to 2.742) | **0.001** |
| Missing | 17 | 16 | 11.6 | 1.447 (0.849 to 2.467) | 0.174 |
| **Subtype** |  |  |  |  |  |
| Acral | 28 | 25 | 10.3 | Reference |  |
| Mucosal | 12 | 12 | 9.7 | 1.068 (0.535 to 2.130) | 0.852 |
| CSD | 34 | 28 | 12.5 | 0.787 (0.458 to 1.351) | 0.384 |
| Non-CSD | 104 | 88 | 10.8 | 0.912 (0.584 to 1.423) | 0.684 |
| **Tumor stage** |  |  |  |  |  |
| Stage III | 30 | 23 | 18.8 | Reference |  |
| Stage IV | 148 | 130 | 10.4 | 1.779 (1.137 to 2.784) | **0.012** |
| **Number of metastatic sites** |  |  |  |  |  |
| ＜3 | 108 | 88 | 13.5 | Reference |  |
| ≥3 | 70 | 65 | 8.4 | 1.868 (1.343 to 2.597) | **<0.001** |
| **Brain metastasis** |  |  |  |  |  |
| No | 137 | 116 | 12.5 | Reference |  |
| Yes | 41 | 37 | 7.9 | 1.518 (1.047 to 2.201) | **0.028** |
| **Liver metastasis** |  |  |  |  |  |
| No | 132 | 112 | 12.2 | Reference |  |
| Yes | 46 | 41 | 9.4 | 1.267 (0.885 to 1.814) | 0.195 |
| **Lung metastasis** |  |  |  |  |  |
| No | 85 | 69 | 13.9 | Reference |  |
| Yes | 93 | 84 | 10.1 | 1.485 (1.076 to 2.049) | **0.016** |
| **Treatment** |  |  |  |  |  |
| D+T | 51 | 44 | 11.1 | Reference |  |
| D+T+aPD-1 | 43 | 30 | 21.9 | 0.391 (0.243 to 0.628) | **<0.001** |
| V | 45 | 45 | 6.2 | 3.227 (2.068 to 5.034) | **<0.001** |
| V+aPD-1 | 39 | 34 | 15 | 0.517 (0.321 to 0.833) | **0.007** |

P values <0.05 in bold are statistically significant.

mPFS, median progression free survival; HR, hazard ratio; ECOG PS, Eastern Cooperative Oncology Group Performance Status; LDH, lactate dehydrogenases; CSD, chronic sun-induced damage; D, dabrafenib; T, trametinib; V, vemurafenib; aPD-1, anti-PD-1 antibody.

**Table S6. Univariable analysis of prognostic factors correlated with OS.**

| **Characteristics** | **Total** | **Events** | **mOS (months)** | **HR (95%CI)** | **P value** |
| --- | --- | --- | --- | --- | --- |
|  |  |  |  |  |  |
| **Age** |  |  |  |  |  |
| ＜60 | 141 | 80 | 26.2 | Reference |  |
| ≥60 | 37 | 21 | 20.7 | 1.176 (0.726 to 1.903) | 0.510 |
| **Gender** |  |  |  |  |  |
| Male | 76 | 44 | 22.6 | Reference |  |
| Female | 102 | 57 | 26.2 | 0.919 (0.620 to 1.362) | 0.672 |
| **ECOG PS** |  |  |  |  |  |
| 0 | 150 | 79 | 29.9 | Reference |  |
| 1-2 | 28 | 22 | 9.6 | 2.440 (1.517 to 3.924) | **<0.001** |
| **Serum LDH** |  |  |  |  |  |
| Normal | 120 | 61 | 38.5 | Reference |  |
| Elevated | 41 | 28 | 14.4 | 2.293 (1.462 to 3.597) | **0.001** |
| Missing | 17 | 12 | 23.4 | 1.777 (0.954 to 3.312) | 0.070 |
| **Subtype** |  |  |  |  |  |
| Acral | 28 | 18 | 16.6 | Reference |  |
| Mucosal | 12 | 8 | 23.4 | 0.940 (0.408 to 2.165) | 0.884 |
| CSD | 34 | 16 | 38.5 | 0.558 (0.284 to 1.096) | 0.09 |
| Non-CSD | 104 | 59 | 23.5 | 0.835 (0.492 to 1.416) | 0.503 |
| **Tumor stage** |  |  |  |  |  |
| Stage III | 30 | 12 | 46.4 | Reference |  |
| Stage IV | 148 | 89 | 22 | 1.997 (1.080 to 3.619) | **0.027** |
| **Number of metastatic sites** |  |  |  |  |  |
| ＜3 | 108 | 49 | 42 | Reference |  |
| ≥3 | 70 | 52 | 16.6 | 2.538 (1.706 to 3.774) | **<0.001** |
| **Brain metastasis** |  |  |  |  |  |
| No | 137 | 74 | 29.9 | Reference |  |
| Yes | 41 | 27 | 14.4 | 1.705 (1.095 to 2.655) | **0.018** |
| **Liver metastasis** |  |  |  |  |  |
| No | 132 | 71 | 29.5 | Reference |  |
| Yes | 46 | 30 | 19.2 | 1.663 (1.082 to 2.556) | **0.020** |
| **Lung metastasis** |  |  |  |  |  |
| No | 85 | 40 | 35.4 | Reference |  |
| Yes | 93 | 61 | 21.2 | 1.651 (1.107 to 2.462) | **0.014** |
| **Treatment** |  |  |  |  |  |
| D+T | 51 | 24 | 32.6 | Reference |  |
| D+T+aPD-1 | 43 | 14 | NR | 0.473 (0.244 to 0.917) | **0.027** |
| V | 45 | 41 | 9.6 | 3.590 (2.158 to 5.973) | **<0.001** |
| V+aPD-1 | 39 | 22 | 40.3 | 0.781 (0.433 to 1.408) | 0.412 |

P values <0.05 in bold are statistically significant.

mOS, median overall survival; HR, hazard ratio; ECOG PS, Eastern Cooperative Oncology Group Performance Status; LDH, lactate dehydrogenases; CSD, chronic sun-induced damage; D, dabrafenib; T, trametinib; V, vemurafenib; aPD-1, anti-PD-1 antibody.

**Table S7. Multivariable analyses of prognostic factors correlated with PFS and OS.**

| **Characteristics** | **PFS** | |  | **OS** | |
| --- | --- | --- | --- | --- | --- |
|  | **HR (95%CI)** | **P value** |  | **HR (95%CI)** | **P value** |
| **ECOG PS (0 vs 1-2)** | 1.670 (1.015 to 2.748) | **0.044** |  | 2.020 (1.131 to 3.607) | **0.017** |
| **Serum LDH (normal vs elevated)** | 1.550 (1.021 to 2.354) | **0.04** |  | 2.071 (1.241 to 3.456) | **0.005** |
| **Tumor stage (stage III vs stage IV)** | 1.528 (0.901 to 2.591) | 0.115 |  | 1.427 (0.708 to 2.876) | 0.320 |
| **Number of metastatic sites (**＜**3 vs** ≥**3)** | 1.181 (0.777 to 1.794) | 0.436 |  | 1.404 (0.848 to 2.323) | 0.187 |
| **Brain metastasis (no vs yes)** | 1.304 (0.840 to 2.025) | 0.237 |  | 1.207 (0.691 to 2.106) | 0.509 |
| **Liver metastasis (no vs yes)** | 1.068 (0.691 to 1.651) | 0.766 |  | 1.432 (0.840 to 2.441) | 0.187 |
| **Lung metastasis (no vs yes)** | 0.882 (0.595 to 1.307) | 0.531 |  | 0.952 (0.589 to 1.539) | 0.842 |
| **Treatment** |  |  |  |  |  |
| **D+T** | Reference |  |  | Reference |  |
| **D+T+aPD-1** | 0.329 (0.200 to 0.543) | **<0.001** |  | 0.378 (0.190 to 0.751) | **0.005** |
| **V** | 3.129 (1.967 to 4.979) | **<0.001** |  | 4.112 (2.418 to 6.992) | **<0.001** |
| **V+aPD-1** | 0.537 (0.330 to 0.875) | **0.013** |  | 0.867 (0.474 to 1.585 | 0.643 |

P values <0.05 in bold are statistically significant.

PFS, progression free survival; OS, overall survival; HR, hazard ratio; ECOG PS, Eastern Cooperative Oncology Group Performance Status; LDH, lactate dehydrogenases; D, dabrafenib; T, trametinib; V, vemurafenib; aPD-1, anti-PD-1 antibody.

**Table S8. Treatment-related adverse events of patients stratified by first-line therapy.**

| **Event, n (%)** | **V (n=45)** | |  | **D+T (n=51)** | |  | **V+aPD-1 (n=39)** | |  | **D+T+aPD-1 (n=43)** | |
| --- | --- | --- | --- | --- | --- | --- | --- | --- | --- | --- | --- |
|  | **Any grade** | **Grade 3-5** |  | **Any grade** | **Grade 3-5** |  | **Any grade** | **Grade 3-5** |  | **Any grade** | **Grade 3-5** |
| **Any adverse event** | **33 (73.3)** | **11 (24.4)** |  | **31 (72.5)** | **11 (21.6)** |  | **38 (97.4)** | **18 (46.2)** |  | **41 (95.3)** | **19 (44.2)** |
| Rash | 18 (40.0) | 6 (13.3) |  | 9 (17.6) | 2 (3.9) |  | 21 (53.8) | 7 (17.9) |  | 23 (53.5) | 7 (16.3) |
| Palmo–plantar erythrodysesthesia | 1 (2.2) | 1 (2.2) |  | 0 | 0 |  | 0 | 0 |  | 0 | 0 |
| Alopecia | 2 (4.4) | 0 |  | 2 (3.9) | 0 |  | 2 (5.1) | 0 |  | 0 | 0 |
| Pruritus | 5 (11.1) | 0 |  | 5 (9.8) | 0 |  | 11 (28.2) | 2 (5.1) |  | 12 (27.9) | 2 (4.7) |
| Photosensitivity reaction | 3 (6.7) | 0 |  | 1 (2.0) | 0 |  | 2 (5.1) | 0 |  | 1 (2.3) | 0 |
| Keratocanthoma | 3 (6.7) | 0 |  | 2 (3.9) | 0 |  | 10 (25.6) | 1 (2.6) |  | 2 (4.7) | 0 |
| Panniculitis | 1 (2.2) | 0 |  | 9 | 1 (2.0) |  | 0 | 0 |  | 10 (23.3) | 1 (2.3) |
| Hand-Foot Syndrome | 3 (6.7) | 1 (2.2) |  | 1 (2.0) | 1 (2.0) |  | 5 (12.8) | 1 (2.6) |  | 1 (2.3) | 0 |
| Vitiligo | 0 | 0 |  | 1 (2.0) | 0 |  | 1 (2.6) | 0 |  | 2 (4.7) | 0 |
| Dermatitis acneiform | 1 (2.2) | 0 |  | 2 (3.9) | 0 |  | 3 (7.7) | 0 |  | 1 (2.3) | 0 |
| Dry skin | 2 (4.4) | 0 |  | 1 (2.0) | 0 |  | 1 (2.6) | 0 |  | 0 | 0 |
| Arthralgia | 15 (33.3) | 2 (4.4) |  | 8 (15.7) | 1 (2.0) |  | 27 (69.2) | 5 (12.8) |  | 6 (14.0) | 2 (4.7) |
| Myalgia | 5 (11.1) | 0 |  | 5 (9.8) | 1 (2.0) |  | 4 (10.3) | 1 (2.6) |  | 1 (2.3) | 0 |
| dorsalgia | 1 (2.2) | 0 |  | 1 (2.0) | 0 |  | 0 | 0 |  | 0 | 0 |
| Diarrhea | 3 (6.7) | 0 |  | 0 | 0 |  | 0 | 0 |  | 3 (7.0) | 0 |
| Nausea | 0 | 0 |  | 2 (3.9) | 0 |  | 1 (2.6) | 0 |  | 0 | 0 |
| Vomiting | 1 (2.2) | 0 |  | 2 (3.9) | 0 |  | 1 (2.6) | 0 |  | 0 | 0 |
| Abdominal pain | 1 (2.2) | 0 |  | 3 (5.9) | 0 |  | 1 (2.6) | 0 |  | 2 (4.7) | 0 |
| Constipation | 0 | 0 |  | 0 | 0 |  | 0 | 0 |  | 1 (2.3) | 0 |
| Decreased appetite | 0 | 0 |  | 0 | 0 |  | 2 (5.1) | 0 |  | 0 | 0 |
| Fatigue | 0 | 0 |  | 5 (9.8) | 0 |  | 5 (12.8) | 0 |  | 5 (11.6) | 0 |
| Pyrexia | 3 (6.7) | 1 (2.2) |  | 22 (43.1) | 2 (3.9) |  | 7 (17.9) | 1 (2.6) |  | 24 (55.8) | 7 (16.3) |
| Headache | 1 (2.2) | 0 |  | 2 (3.9) | 1 (2.0) |  | 3 (7.7) | 0 |  | 1 (2.3) | 0 |
| Dizziness | 1 (2.2) | 0 |  | 4 (7.8) | 1 (2.0) |  | 0 | 0 |  | 0 | 0 |
| Chills | 0 | 0 |  | 0 | 0 |  | 1 (2.6) | 0 |  | 4 (9.3) | 0 |
| Anaemia | 0 | 0 |  | 0 | 0 |  | 1 (2.6) | 0 |  | 1 (2.3) | 0 |
| ALT increased | 4 (8.9) | 2 (4.4) |  | 3 (5.9) | 2 (3.9) |  | 8 (20.5) | 2 (5.1) |  | 5 (11.6) | 2 (4.7) |
| AST increased | 3 (6.7) | 1 (2.2) |  | 3 (5.9) | 2 (3.9) |  | 8 (20.5) | 2 (5.1) |  | 4 (9.3) | 1 (2.3) |
| Increased bilirubin | 1 (2.2) | 0 |  | 1 (2.0) | 0 |  | 4 (10.3) | 1 (2.6) |  | 1 (2.3) | 0 |
| Increased blood creatinine | 1 (2.2) | 1 (2.2) |  | 0 | 0 |  | 1 (2.6) | 1 (2.6) |  | 0 | 0 |
| Hyperglycemia | 0 | 0 |  | 2 (3.9) | 1 (2.0) |  | 0 | 0 |  | 0 | 0 |
| GGT increased | 0 | 0 |  | 2 (3.9) | 1 (2.0) |  | 0 | 0 |  | 0 | 0 |
| ALP increased | 1 (2.2) | 1 (2.2) |  | 1 (2.0) | 1 (2.0) |  | 1 (2.6) | 1 (2.6) |  | 1 (2.3) | 0 |
| CK increased | 0 | 0 |  | 0 | 0 |  | 1 (2.6) | 1 (2.6) |  | 1 (2.3) | 0 |
| Lipase increased | 0 | 0 |  | 2 (3.9) | 1 (2.0) |  | 0 | 0 |  | 3 (7.0) | 0 |
| Amylase increased | 0 | 0 |  | 2 (3.9) | 1 (2.0) |  | 0 | 0 |  | 1 (2.3) | 0 |
| Lymphopenia | 0 | 0 |  | 1 (2.0) | 0 |  | 0 | 0 |  | 1 (2.3) | 1 (2.3) |
| Cough | 0 | 0 |  | 0 | 0 |  | 3 (7.7) | 0 |  | 2 (4.7) | 0 |
| Pneumonia | 0 | 0 |  | 0 | 0 |  | 3 (7.7) | 2 (5.1) |  | 1 (2.3) | 1 (2.3) |
| Hypothyroidism | 0 | 0 |  | 0 | 0 |  | 4 (10.3) | 0 |  | 3 (7.0) | 0 |
| Hyperthyroidism | 0 | 0 |  | 0 | 0 |  | 2 (5.1) | 0 |  | 0 | 0 |
| Adrenal cortical hypofunction | 0 | 0 |  | 0 | 0 |  | 2 (5.1) | 0 |  | 2 (4.7) | 0 |
| Thyroiditis | 0 | 0 |  | 0 | 0 |  | 1 (2.6) | 0 |  | 0 | 0 |
| Myocarditis | 0 | 0 |  | 0 | 0 |  | 1 (2.6) | 1 (2.6) |  | 3 (7.0) | 2 (4.7) |
| Hypertension | 0 | 0 |  | 0 | 0 |  | 0 | 0 |  | 1 (2.3) | 0 |
| Uveitis | 0 | 0 |  | 0 | 0 |  | 3 (7.7) | 0 |  | 3 (7.0) | 2 (4.7) |
| Chorioretinopathy | 0 | 0 |  | 0 | 0 |  | 1 (2.6) | 0 |  | 2 (4.7) | 2 (4.7) |

V, vemurafenib; D, dabrafenib; T, trametinib; aPD-1, anti-PD-1 antibody; TrAEs, treatment-related adverse events; ALT, alanine aminotransferase; AST, aspartate transaminase; GGT, gamma-glutamyltransferase; ALP, alkaline phosphatase; CK, creatine kinase.

**Table S9. Adverse events leading to dose reduction, dose interruption and treatment discontinuation.**

| **Event, n (%)** | **V (n=45)** | **D+T (n=51)** | **V+aPD-1 (n=39)** | **D+T+aPD-1 (n=43)** |
| --- | --- | --- | --- | --- |
| **Dose reduction** | 7 (15.6) | 1 (2.0) | 15 (38.5) | 1 (2.3) |
| **Dose interruption** | 5 (11.1) | 8 (15.7) | 11 (28.2) | 12 (30.2) |
| **Treatment discontinuation** | 1 (2.2) | 1 (2.0) | 1 (2.6) | 3 (7.0) |

V, vemurafenib; D, dabrafenib; T, trametinib; aPD-1, anti-PD-1 antibody.

**Table S10. Immune-mediated adverse events occurring in at least one patient in either treatment arm.**

| **Event, n (%)** | **V+aPD-1 (n=39)** | |  | **D+T+aPD-1 (n=43)** | |
| --- | --- | --- | --- | --- | --- |
|  | **Any grade** | **Grade 3-5** |  | **Any grade** | **Grade 3-5** |
| **At least one immune-mediated adverse event** | **25 (64.1)** | **7 (17.9)** |  | **30 (69.8)** | **8 (18.6)** |
| Severe skin reactions | 16 (41.0) | 3 (7.7) |  | 14 (32.6) | 2 (4.7) |
| Pyrexia | 16 (41.0) | 1 (2.6) |  | 19 (44.2) | 1 (2.3) |
| Pneumonitis | 3 (7.7) | 2 (5.1) |  | 1 (2.3) | 1 (2.3) |
| Hepatitis | 5 (12.8) | 1 (2.6) |  | 3 (7.0) | 0 |
| Hypothyroidism | 4 (10.3) | 0 |  | 3 (7.0) | 0 |
| Hyperthyroidism | 2 (5.1) | 0 |  | 0 | 0 |
| Uveitis | 3 (7.7) | 0 |  | 4 (9.3) | 3 (7.0) |
| Hypophysitis | 2 (5.1) | 0 |  | 2 (4.7) | 0 |
| Pancreatitis | 0 | 0 |  | 1 (2.3) | 0 |
| Myocarditis | 1 (2.6) | 1 (2.6) |  | 3 (7.0) | 2 (4.7) |
| Gastroenteritis | 1 (2.6) | 0 |  | 2 (4.7) | 0 |

V, vemurafenib; D, dabrafenib; T, trametinib; aPD-1, anti-PD-1 antibody.
